# Two novel mouse models of slow-wave-sleep enhancement in aging and Alzheimer’s disease

**DOI:** 10.1093/sleepadvances/zpac022

**Published:** 2022-06-30

**Authors:** Oghomwen E Ogbeide-Latario, Loris L Ferrari, Heinrich S Gompf, Christelle Anaclet

**Affiliations:** Department of Neurobiology, University of Massachusetts Chan Medical School, Worcester, MA, USA; Morningside Graduate School of Biomedical Sciences, University of Massachusetts Chan Medical School, Worcester, MA, USA; Department of Neurobiology, University of Massachusetts Chan Medical School, Worcester, MA, USA; Department of Neurobiology, University of Massachusetts Chan Medical School, Worcester, MA, USA; Department of Neurological Surgery, University of California Davis School of Medicine, Davis CA, USA; Department of Neurobiology, University of Massachusetts Chan Medical School, Worcester, MA, USA; Department of Neurological Surgery, University of California Davis School of Medicine, Davis CA, USA

**Keywords:** slow-wave-sleep (SWS) enhancement, slow wave activity (SWA), aging, Alzheimer’s disease (AD), mouse models, chemogenetics, clozapine-N-oxide (CNO), parafacial zone (PZ), APP/PS1 mice

## Abstract

Aging and Alzheimer’s disease (AD) are both associated with reduced quantity and quality of the deepest stage of sleep, called slow-wave-sleep (SWS). Slow-wave-sleep deficits have been shown to worsen AD symptoms and prevent healthy aging. However, the mechanism remains poorly understood due to the lack of animal models in which SWS can be specifically manipulated. Notably, a mouse model of SWS enhancement has been recently developed in adult mice. As a prelude to studies assessing the impact of SWS enhancement on aging and neurodegeneration, we first asked whether SWS can be enhanced in animal models of aging and AD.

The chemogenetic receptor hM3Dq was conditionally expressed in GABAergic neurons of the parafacial zone of aged mice and AD (APP/PS1) mouse model. Sleep–wake phenotypes were analyzed in baseline condition and following clozapine-*N*-oxide (CNO) and vehicle injections. Both aged and AD mice display deficits in sleep quality, characterized by decreased slow wave activity. Both aged and AD mice show SWS enhancement following CNO injection, characterized by a shorter SWS latency, increased SWS amount and consolidation, and enhanced slow wave activity, compared with vehicle injection. Importantly, the SWS enhancement phenotypes in aged and APP/PS1 model mice are comparable to those seen in adult and littermate wild-type mice, respectively. These mouse models will allow investigation of the role of SWS in aging and AD, using, for the first time, gain-of SWS experiments.

**Statement of Significance**

Deficits of sleep and specifically in deep sleep, called slow-wave-sleep, are now recognized to have serious consequences on health. Of note, aging and Alzheimer’s disease (AD) are associated with a decreased proportion of slow-wave-sleep which could further amplify symptoms. Unfortunately, the mechanism by which slow-wave-sleep promotes healthy aging remains poorly understood due to the lack of animal models. In this study, we validate aging and AD mouse models in which slow-wave-sleep can be triggered and enhanced on demand. These mouse models will permit to study mechanically the role of slow-wave-sleep in aging and AD.

## Introduction

A growing number of studies have suggested a major role of the deepest stage of sleep, called slow-wave-sleep (SWS) in pathologies associated with aging [1] and neurodegenerative diseases such as Alzheimer’s disease (AD) [2, 3]. Aging and AD are associated with decreased amounts of deep sleep, sleep fragmentation and circadian disruption of the sleep–wake cycle [4–6]. Sleep disruption has also been shown to be a risk factor in all-cause mortality [7] and specifically in AD progression [8]. However, the mechanism by which sleep promotes optimal health remains largely elusive, due to lack of tools to modulate specific sleep stages [9].

Understanding these mechanisms is especially important given the observed sleep deficits in numerous diseases. Multiple recent studies explore the beneficial role of sleep enhancement in physiology and disease, suggesting that sleep could be an easily modifiable and treatable risk factor to forestall AD and promote healthy aging [10–12]. However, the mechanisms by which sleep in general and SWS deficits in particular affect AD symptoms and aging quality remain poorly understood. This is of high importance given that the population of individuals 65 years and older is increasing and expected to double over the next 25 years, estimating one in five people over the age of 65 in the United States alone [13]. With the predicted rise in the aging population [14], interventional strategies to preserve optimal physiological functions is critical to sustain excellent quality of life and reduce both health and economic burdens. Therefore, understanding the mechanism by which sleep promotes physiological homeostasis is critical.

Two major sleep stages are distinguished, rapid eye movement (REM) sleep and non-REM (NREM) sleep. In humans, NREM sleep includes 3 substages, 1–3, corresponding to increased sleep depth. Stage 3, the deepest NREM sleep stage, is also called SWS. In rodents, only one NREM sleep stage is distinguished, the depth of NREM sleep being measured by the power of the cortical electroencephalogram (EEG) slow frequencies (delta band, 0.5–4 Hz), also called slow wave activity (SWA). Recent studies have suggested that deep NREM sleep, SWS, is a major player in modulating other physiological functions [12, 15]. However, studying the specific role of SWS has been challenging due to the difficulty to specifically manipulate this sleep stage.

In previous studies, we have developed a mouse model of SWS enhancement in adult mice [16, 17] using chemogenetic activation of the sleep-promoting parafacial zone GABAergic neurons (PZ^GABA^), a new sleep-promoting neuronal population. The sleep-active phenotype of PZ^GABA^ was confirmed in a separate study, in vivo electrophysiological recordings in freely moving rats show that the PZ contains neurons specifically active during NREM sleep [18]. Moreover, this study showed that PZ sleep-active neurons start discharging at sleep onset and therefore seem to be more involved in NREM sleep maintenance rather than in NREM sleep induction. In contrast, when neurons were recorded in the parvocellular reticular nucleus part alpha (PCRtA), located just ventral and lateral from PZ, no sleep-active neurons were found [19]. The paucity of sleep-active neurons recorded in this particular study by Sakai (2017) is consistent with our observations that chemogenetic receptor expression in this area (the mis-injections of the viral vector that serve as our anatomical controls) does not result in an enhanced sleep phenotype. We showed that chemogenetic activation of parafacial zone GABAergic neurons (PZ^GABA^) induces long lasting episodes (3–6 h) of NREM sleep characterized by enhanced SWA, indistinguishable from SWS [16]. These phenotypes are unique in that other mouse models in which NREM sleep is enriched simply show increased NREM sleep amounts not always associated with enhanced SWA. Our mouse model of SWS enhancement is also unique because this is the first in which SWS can be triggered and enhanced on demand, and sustained for a long durations (3–6 h). Finally, in our mouse model, REM sleep is inhibited during SWS enhancement in contrast to other mouse models of NREM sleep enrichment in which REM sleep amount is increased in association with NREM sleep increase. Therefore, chemogenetic activation of PZ^GABA^ allows interrogation of the role of deep sleep, SWS, in physiology and diseases, using, for the first time, gain-of-SWS experiments.

Studies in humans have suggested that in aging and AD, reduced sleep quality could be due to the loss of sleep promoting neurons [20] and/or neuronal loss in the cortex [21, 22]. Therefore, in this study, we ask whether chemogenetic activation of PZ^GABA^ can enhance SWS in aged and AD mice to the same extent as in adult and littermate control mice, respectively. Our findings show that chemogenetic activation of PZ^GABA^ results in identical phenotypes in aged, AD and adult mice, providing mouse models to study the role of sleep in age- and AD-related deficits.

## Materials and Methods

### Animals

A total of 98 pathogen-free mice, on the C57BL/6J genetic background, were used in this study. To study the effect of age, 17 adult (3–6 months; 26–42 g), and 17 aged (18–24 months; 27–41 g) male *Vgat-IRES-Cre::EGFP-L10a* (Vgat::GFP) and 8 adult and 9 aged littermate Cre- mice were used. The Vgat::GFP mouse line resulted from the cross between *Vgat*-IRES-Cre (S*lc32a1tm2(cre)Lowl*/J, Jackson stock #028862) and *EGFP-L10A* (Jackson stock #024750). This mouse line expresses Cre recombinase from the Vgat (vesicular GABA/glycine transporter) genomic locus and EGFP (enhanced green fluorescent protein) in a Cre-dependent manner. To study the effect of AD, we generated an APP/PS1/Vgat::GFP mouse line by crossing B6.CgTg(APPswe,PSEN1dE9)85Dbo/Mmjax (Jackson stock #5864) with the Vgat::GFP mice described above. This crossing resulted in a quadruple transgenic mouse model, a Vgat::GFP mouse expressing the mouse/human amyloid precursor protein (Mo/HuAPP695swe) and a mutant human presenilin 1 (PS1-dE9). 9 female (9–12 months, 24–42 g) and 7 male (14–17 months, 32.5–48.5 g) APP/PS1/Vgat::GFP (AP+) mice were included in the study. The littermate Vgat::GFP mice lacking APP/PS1 (AP-) were used as controls (8 females and 7 males). Littermate Cre− mice were used to control for the effect of CNO in mice not expressing hM3Dq receptors (5 males and 4 females Cre−/AP−; 3 males and 4 females Cre−/AP+). Mice were bred at our animal facility and underwent genotyping both before and after experiments, as previously described [23, 24]. Care of these animals met the National Institutes of Health standards, as set forth in the *Guide for the Care and Use of Laboratory Animals* and all protocols were approved by the University of Massachusetts Chan Medical School Institutional Animal Care and Use Committees.

### Surgery

Naïve mice were submitted to two independent surgeries separated by at least two weeks. Mice were anesthetized with ketamine/xylazine [100 and 10 mg/kg, respectively, intraperitoneally (IP)] and then placed in a stereotaxic apparatus. During the first surgery, to selectively express the chemogenetic hM3Dq receptor in GABAergic neurons of the parafacial zone (PZ^GABA^), mice received bilateral injections of an adeno-associated viral (AAV; serotype 2) vector expressing the hM3Dq receptor and mCherry (reporter gene) in a Cre-dependent configuration (hSyn-DIO-hM3Dq-mCherry-AAV, UMASS Vector Core, titer: 3.0E+12 viral particles/mL) into the PZ, as previously described [16]. Coordinates from Bregma were −5.6 mm Antero-posterior, ± 1.0 mm Lateral, −4.2 mm Dorso-ventral, as per the mouse atlas of Paxinos and Franklin [25]. The AAV (200 nL) was injected into the PZ of mice using a 10 µL Hamilton syringe (Hamilton Co., Reno, NV) at a rate of 1 nL/min driven by an UMP2 microinfusion pump with a SMARTouch Controller (World Precision Instruments, Inc., Sarasota, FL). During the second surgery, mice were implanted with four EEG screw electrodes (2 frontal [1 mm frontal, 1 mm lateral from bregma] and 2 parietal [mid-distance between bregma and lambda and 1 mm lateral from the mid-line] electrodes; Pinnacle Technology Inc., Catalog #8403) and two flexible electromyogram (EMG) wire electrodes (in the neck muscles; Plastics One, catalog #E363/76/SPC), previously soldered to a 6-pin connector (Heilind Electronics, catalog #853-43-006-10- 001000) and the assembly was secured to the skull with dental cement. After completing the surgery, mice were kept in a warm environment until resuming normal activity as previously described [16].

### Sleep–wake recording

Following a minimum of 10 days for postsurgical recovery, the mice were housed individually in transparent barrels in an insulated sound-proofed recording chamber maintained at an ambient temperature of 22 ± 1°C and on a 12 h light/dark cycle (lights-on at 07:00, Zeitgeber time: ZT0) with food and water available ad libitum. Mice were connected to flexible recording cables and habituated to the recording conditions for 5 days before starting polygraphic recording. One cortical EEG (bipolar, fronto-parietal, ipsilateral) and the EMG signals were amplified (A-M System 3500, United States) and digitalized with a resolution of 256 Hz using Vital Recorder (Kissei, Japan). Mice were recorded for a 24 h baseline period, followed by IP injections of saline (vehicle injection) or Clozapine-N-oxide (CNO, NIMH Chemical Synthesis and Drug Supply Program; 0.3 mg/kg, 0.003 mg/mL in saline). Injections were performed at 19:00 (ZT12, beginning of the dark period, at a time of high wake-drive), in a randomized cross-over design, with each injection separated by a 2–3 day washout period.

### Sleep scoring and analysis

Using SleepSign for Animal (Kissei, Japan) assisted by spectral analysis using fast Fourier transform (FFT), polygraphic records were visually scored in 10 s epochs for wakefulness, SWS, and REM sleep. Wakefulness is characterized by low amplitude fast frequency EEG associated with EMG activity. SWS is characterized by high amplitude, low frequency EEG, and low EMG activity. REM sleep is characterized by an EEG dominated by hippocampal theta rhythm and no EMG activity. Similar to human scoring rules [26], microarousals were left out of the analysis, only episodes of wakefulness or REM sleep lasting > 10 s and episodes of SWS lasting > 20 s were scored as such. The percentage of time spent in wakefulness, SWS and REM sleep were calculated and summarized for each group and each condition. The SWS and REM sleep latencies are defined as the time between the end of the IP injection and the onset of the first SWS episode, lasting >20 s, and the onset of the first REM sleep episode, lasting >10 s, respectively. Sleep–wake fragmentation was assessed by analyzing the distribution of each vigilance stage in different bout lengths. Vigilance stages were separated into eight bout lengths (< 30, 40–70, 80–150, 160–310, 320–630, 640–1270, 1280–2550, and >2560 s) [27, 28]. For each vigilance stage, the number of episodes and the percentage of the vigilance stages occurring in each bout length were calculated (Tables 1–4). Recordings were scored again in 4 s epochs to allow for performance of the cortical EEG power spectral analysis. Based on visual and spectral analysis, epochs containing artifacts occurring during active wakefulness (with large movement artifacts), containing two vigilance states or containing spontaneous epileptiform discharges [29] were visually identified and omitted from the spectral analysis. Re-scoring with a shorter epoch length allows us to minimize the number of recording epochs omitted from the analysis due to movement artifacts. Recordings containing artifacts more than 20% of the recorded time were removed from the spectral analysis. Cortical EEG power spectra were computed for consecutive 4 s epochs within the frequency range of 0.5–55 Hz using a fast Fourier transform (FFT) routine. The data were collapsed into 0.5 Hz bins. Baseline cortical EEG power spectra were analyzed during a 3-hr period within the light period (10:00–13:00, ZT3-ZT6; sleepy period) and within a 3-hr period in the dark period (19:00–22:00, ZT12-ZT15; active period). The data were standardized by expressing each frequency bin as a percentage relative to the total power of the same epochs [for example, (bin power * 100)/0.5–55 Hz total power]. To analyze the EEG frequency bands, power bins were summed in delta (δ, 0.5–4.5 Hz), theta (θ, 5–9 Hz), sigma (α, 9.5–15 Hz), beta (β, 15.5–30 Hz) and gamma (γ, 30.5–55 Hz) bands. To determine the effect of the injection on cortical EEG power distribution, the FFT data were collected from 10 min after injection (according to our previous study showing that SWS latency is no longer than 10 min after CNO injection [16]) and for 3-h following vehicle injection (saline) and 1- or 3-h following CNO injection, in Vgat-Cre+ and Vgat-Cre− mice, respectively. This allows for FFT analyses over similar amounts of SWS between vehicle and CNO conditions [16]. The data were standardized by expressing each frequency bin as a percentage relative to the same bin under baseline conditions and from the same mouse. To analyze the EEG frequency bands, power bins were summed in delta (δ, 0.5–4.5 Hz), theta (θ, 5–9 Hz), sigma (α, 9.5–15 Hz), beta (β, 15.5–30 Hz) and gamma (γ, 30.5–55 Hz) bands, and expressed as a percentage of the respective baseline power band.

**Table 1:** Number of episodes (± SEM) of wakefulness, slow-wave-sleep, and REM sleep amounts in each bout length to the total amount of wakefulness, slow-wave-sleep, and REM sleep during the dark and light periods in Vgat::GFP adult (*n* = 17) and aged (*n* = 17) mice; and during the 3-hr following vehicle or CNO injection in Vgat::GFP adult (*n* = 8) and aged (*n* = 8) mice. ****p < .0001, aged vs. adult mice; ^**#**^*p* < .05, ^**##**^*p* < .01, ^**###**^*p* <.001, ^**####**^*p* < .0001, dark vs. light period; and **^***p* < .05, **^^^^***p* < .0001 CNO vs. vehicle injection, two-way ANOVA followed by a post hoc Bonferroni test.

Bout length (min)	Wakefulness		Slow-Wave-Sleep		REM Sleep	
Light period	Adult	Aged	Adult	Aged	Adult	Aged
0.1–0.5	78.2 ± 9.5	91.0 ± 8.4	14.7 ± 3.3	12.7 ± 2.0	12.5 ± 2.2	17.2 ± 2.4
0.5–1	13.4 ± 1.3	13.2 ± 1.5	15.8 ± 3.0	20.1 ± 2.0	9.4 ± 0.8	10.6 ± 1.3
1–2.5	5.3 ± 0.8	7.5 ± 1.1	30.2 ± 4.8	43.2 ± 4.3	16.7 ± 1.2	16.9 ± 0.8^**###**^
2.5–5	2.9 ± 0.4	4.2 ± 1.0	26.2 ± 1.8	33.0 ± 2.3	6.7 ± 0.5	1.7 ± 0.3****
5–10	4.3 ± 0.6	5.2 ± 1.0	19.3 ± 1.7	16.5 ± 1.6	0.1 ± 0.1	0.0 ± 0.0
10–20	4.4 ± 0.5	4.5 ± 0.7	5.4 ± 1.0	3.9 ± 0.8	0.0 ± 0.0	0.0 ± 0.0
20–40	2.3 ± 0.3	3.0 ± 0.5	0.12 ± 0.1	0.2 ± 0.1	0.0 ± 0.0	0.0 ± 0.0
40–∞	1.0 ± 0.2	0.7 ± 0.2	0.0 ± 0.0	0.0 ± 0.0	0.0 ± 0.0	0.0 ± 0.0
Dark period						
0.1–0.5	51.5 ± 7.3	72.7 ± 10.3	12.4 ± 2.4	18.7 ± 3.7	10.1 ± 2.1	11.2 ± 2.4
0.5–1	8.2 ± 1.2^**#**^	12.8 ± 1.1	13.7 ± 2.2	21.9 ± 3.6	7.7 ± 0.8	6.9 ± 0.9
1–2.5	5.1 ± 0.6	9.9 ± 2.0	25.5 ± 3.6	38.4 ± 5.3	15.1 ± 1.3	10.5 ± 1.2
2.5–5	6.7 ± 0.9^**##**^	6.9 ± 1.1	23.2 ± 1.6	28.9 ± 2.0	3.9 ± 0.6^**##**^	1.4 ± 0.3
5–10	10.5 ± 1.0^**####**^	8.7 ± 1.2	16.1 ± 1.7	11.8 ± 1.8	0.0 ± 0.0	0.0 ± 0.0
10–20	6.5 ± 0.7	7.1 ± 0.8	2.1 ± 0.6	2.2 ± 0.9	0.0 ± 0.0	0.0 ± 0.0
20–40	3.1 ± 0.4	3.2 ± 0.5	0.0 ± 0.0	0.0 ± 0.0	0.0 ± 0.0	0.0 ± 0.0
40–∞	1.1 ± 0.2	0.8 ± 0.2	0.0 ± 0.0	0.0 ± 0.0	0.0 ± 0.0	0.0 ± 0.0
Control injection						
0.1–0.5	10.4 ± 1.2	11.9 ± 4.4	2.8 ± 0.9	2.5 ± 1.0	2.1 ± 0.8	0.9 ± 0.6
0.5–1	1.1 ± 0.2	3.6 ± 0.7	3.1 ± 1.0	5.8 ± 2.8	1.6 ± 0.5	0.6 ± 0.5
1–2.5	0.9 ± 0.4	3.1 ± 1.2	5.3 ± 0.9	7.5 ± 2.0	2.8 ± 0.7	2.1 ± 0.9
2.5–5	2.0 ± 0.9	2.3 ± 0.9	4.9 ± 1.0	5.9 ± 0.7	0.6 ± 0.3	0.1 ± 0.1
5–10	2.5 ± 0.5	1.1 ± 0.4	4.1 ± 0.7	2.0 ± 0.7	0.0 ± 0.0	0.0 ± 0.0
10–20	2.0 ± 0.8	1.8 ± 0.4	0.4 ± 0.2	1.1 ± 0.6	0.0 ± 0.0	0.0 ± 0.0
20–40	1.3 ± 0.5	0.9 ± 0.2	0.0 ± 0.0	0.0 ± 0.0	0.0 ± 0.0	0.0 ± 0.0
40–∞	0.1 ± 0.1	0.6 ± 0.3	0.0 ± 0.0	0.0 ± 0.0	0.0 ± 0.0	0.0 ± 0.0
**CNO 0.3 mg/kg**						
0.1–0.5	6.9 ± 2.0	9.0 ± 1.1	3.8 ± 0.9	2.6 ± 0.7	0.0 ± 0.0	0.5 ± 0.4
0.5–1	1.0 ± 0.4	3.6 ± 0.7	2.3 ± 0.5	4.6 ± 1.4	0.0 ± 0.0	0.1 ± 0.1
1–2.5	1.6 ± 0.5	2.8 ± 0.8	2.1 ± 1.0	4.5 ± 1.4	0.1 ± 0.1**^**	0.1 ± 0.1
2.5–5	1.8 ± 0.6	2.5 ± 1.0	1.3 ± 0.5	2.3 ± 0.7**^**	0.0 ± 0.0	0.0 ± 0.0
5–10	1.4 ± 0.3	0.9 ± 0.4	1.4 ± 0.5	3.0 ± 0.6	0.0 ± 0.0	0.0 ± 0.0
10–20	0.9 ± 0.4	1.0 ± 0.3	0.8 ± 0.4	0.9 ± 0.3	0.0 ± 0.0	0.0 ± 0.0
20–40	0.3 ± 0.3	0.3 ± 0.3	1.0 ± 0.4	1.3 ± 0.3**^**	0.0 ± 0.0	0.0 ± 0.0
40–∞	0.0 ± 0.0	0.0 ± 0.0	1.0 ± 0.0**^^^^**	0.8 ± 0.2**^**	0.0 ± 0.0	0.0 ± 0.0

**Table 2:** Percentage (±SEM) of wakefulness, slow-wave-sleep, and REM sleep amounts in each bout length to the total amount of wakefulness, slow-wave-sleep, and REM sleep during the light and dark periods in Vgat::GFP adult (*n* = 17) and aged (*n* = 17) mice; and during the 3-hr following vehicle or CNO injection in Vgat::GFP adult (*n* = 8) and aged (*n* = 8) mice. *p < .05, **p < .01, ***p < .001, ****p < .0001, aged vs. adult mice; ^**##**^*p* < .01, dark vs. light period; and ^*p* < .05, **^^***p* < .01, CNO vs. vehicle injection, two-way ANOVA followed by a post hoc Bonferroni test.

Bout length (min)	Wakefulness		Slow-Wave-Sleep		REM Sleep	
Light phase	Adult	Aged	Adult	Aged	Adult	Aged
< 0.5	8.9 ± 1.1	8.9 ± 0.8	1.5 ± 0.3	1.3 ± 0.2	6.9 ± 1.0	11.9 ± 1.2*
0.5–1	4.0 ± 0.4	3.9 ± 0.6	3.6 ± 0.7	4.4 ± 0.5	11.8 ± 0.9	17.3 ± 1.5*
1–2.5	3.1 ± 0.4	4.7 ± 1.0	14.7 ± 2.3	20.5 ± 2.1	45.9 ± 2.5	61.2 ± 2.5**
2.5–5	4.5 ± 0.7	6.0 ± 1.7	26.1 ± 2.1	31.5 ± 2.4	34.2 ± 3.3	9.6 ± 2.0****
5–10	11.0 ± 1.4	13.8 ± 3.0	35.7 ± 2.7	29.0 ± 2.5	1.2 ± 0.8	0.0 ± 0.0
10–20	23.7 ± 3.1	23.1 ± 3.3	17.8 ± 3.0	12.6 ± 2.4	0.0 ± 0.0	0.0 ± 0.0
20–40	24.9 ± 3.6	27.2 ± 4.3	0.6 ± 0.4	0.9 ± 0.6	0.0 ± 0.0	0.0 ± 0.0
40–∞	19.9 ± 4.7	12.4 ± 3.5	0.0 ± 0.0	0.0 ± 0.0	0.0 ± 0.0	0.0 ± 0.0
Dark Phase						
<0.5	4.2 ± 0.6^**##**^	5.7 ± 0.8	1.7 ± 0.3	3.0 ± 0.6	7.2 ± 1.3	10.9 ± 1.7
0.5–1	1.9 ± 0.3^**##**^	3.3 ± 0.7	4.1 ± 0.7	7.4 ± 1.3	13.4 ± 1.1	18.0 ± 1.9
1–2.5	2.4 ± 0.3	5.0 ± 1.1	16.1 ± 2.3	25.3 ± 3.4	55.2 ± 2.9	59.3 ± 3.8
2.5–5	7.0 ± 0.8	8.1 ± 2.0	31.6 ± 2.2	34.8 ± 2.3	21.6 ± 2.9	11.9 ± 2.5
5–10	20.8 ± 2.2^**##**^	17.6 ± 2.2	38.1 ± 3.0	22.9 ± 3.7	0.0 ± 0.0	0.0 ± 0.0
10–20	25.3 ± 2.5	26.6 ± 3.5	8.5 ± 2.4	6.6 ± 2.5*	0.0 ± 0.0	0.0 ± 0.0
20–40	20.6 ± 3.4	23.2 ± 3.4	0.0 ± 0.0	0.0 ± 0.0	0.0 ± 0.0	0.0 ± 0.0
40–∞	17.8 ± 3.7	10.5 ± 2.6	0.0 ± 0.0	0.0 ± 0.0	0.0 ± 0.0	0.0 ± 0.0
Vehicle injection						
<0.5	3.2 ± 0.6	11.9 ± 4.4	1.8 ± 0.7	1.2 ± 0.4	9.9 ± 2.9	4.7 ± 3.1
0.5–1	1.0 ± 0.2	3.6 ± 0.7	5.8 ± 2.8	6.8 ± 2.7	15.3 ± 5.6	8.8 ± 6.1
1–2.5	1.8 ± 0.7	3.1 ± 1.2	15.9 ± 3.1	20.1 ± 4.6	56.5 ± 9.2	58.2 ± 14.1
2.5–5	8.6 ± 3.8	2.3 ± 0.9	29.2 ± 4.1	38.8 ± 5.0	18.3 ± 7.6	3.3 ± 3.3
5–10	21.3 ± 5.5	1.1 ± 0.4	40.6 ± 6.8	15.3 ± 5.3***	0.0 ± 0.0	0.0 ± 0.0
10–20	27.0 ± 9.3	1.8 ± 0.4	6.8 ± 3.6	17.8 ± 8.8	0.0 ± 0.0	0.0 ± 0.0
20–40	31.5 ± 10.4	0.9 ± 0.2	0.0 ± 0.0	0.0 ± 0.0	0.0 ± 0.0	0.0 ± 0.0
40–∞	5.6 ± 5.6	0.6 ± 0.3	0.0 ± 0.0	0.0 ± 0.0	0.0 ± 0.0	0.0 ± 0.0
CNO 0.3 mg/kg						
<0.5	6.9 ± 2.0	8.8 ± 2.4	1.3 ± 0.4	0.7 ± 0.2	0.0 ± 0.0	17.1 ± 12.7
0.5–1	2.1 ± 1.1	8.4 ± 2.5	1.3 ± 0.3	3.4 ± 1.5	0.0 ± 0.0	2.6 ± 2.6
1–2.5	9.6 ± 4.6	9.8 ± 3.2	2.7 ± 1.2**^**	6.2 ± 2.2	12.5 ± 12.5**^**	5.3 ± 5.3
2.5–5	24.2 ± 11.5	15.1 ± 6.2	3.3 ± 1.1**^^**	5.6 ± 1.7**^^**	0.0 ± 0.0	0.0 ± 0.0
5–10	30.1 ± 8.5	21.4 ± 11.2	8.9 ± 3.0**^**	16.7 ± 4.2	0.0 ± 0.0	0.0 ± 0.0
10–20	20.6 ± 8.6	31.7 ± 8.7	5.7 ± 3.0	9.2 ± 3.1	0.0 ± 0.0	0.0 ± 0.0
20–40	6.7 ± 6.7	4.9 ± 4.9	18.5 ± 7.6	24.6 ± 5.0**^**	0.0 ± 0.0	0.0 ± 0.0
40–∞	0.0 ± 0.0	0.0 ± 0.0	58.4 ± 8.1**^^**	33.7 ± 7.8**^**	0.0 ± 0.0	0.0 ± 0.0

**Table 3:** Number of episodes (± SEM) of wakefulness, slow-wave-sleep, and REM sleep amounts in each bout length to the total amount of wakefulness, slow-wave-sleep, and REM sleep during the light and dark periods: AP+ (*n* = 15) and AP− (*n* = 16) mice; and during the 3-hr post control and CNO injection AP+ (*n* =10) and AP− (*n* = 11) mice. No significant difference, AP+ vs. AP−; ^**#**^*p* < .05, ^**##**^*p* < .01, ^**###**^*p* < .001, ^**####**^*p* < .0001, dark vs. light period; and **^***p* < .05, **^^***p* < .01, **^^^***p* < .001, **^^^^***p* < .0001 CNO vs. vehicle injection, two-way ANOVA followed by a post hoc Bonferroni test.

Bout length (min)	Wakefulness		Slow-Wave-Sleep		REM Sleep	
Light Phase	AP-	AP+	AP-	AP+	AP-	AP+
<0.5	55.7 ± 3.2	53.7 ± 2.9	4.4 ± 0.6	7.1 ± 0.9	12.9 ± 1.6	11.7 ± 1.2
0.5–1	14.5 ± 1.7	12.4 ± 1.1	8.2 ± 0.7	9.7 ± 0.9	10.1 ± 0.9	8.4 ± 0.7
1–2.5	7.4 ± 1.2	6.7 ± 1.1	21.8 ± 2.7	20.9 ± 2.0	15.7 ± 1.0	14.8 ± 1.0
2.5–5	2.9 ± 0.6	4.9 ± 0.9	27.2 ± 2.3	27.5 ± 1.7	1.6 ± 0.4	3.1 ± 0.5
5–10	4.4 ± 0.8	6.5 ± 0.8	24.9 ± 1.1	23.2 ± 1.4	0.0 ± 0.0	0.0 ± 0.0
10–20	4.1 ± 0.5	5.8 ± 0.6	6.1 ± 0.9	4.9 ± 0.8	0.0 ± 0.0	0.0 ± 0.0
20–40	2.5 ± 0.3	2.3 ± 0.3	0.1 ± 0.1	0.1 ± 0.1	0.0 ± 0.0	0.0 ± 0.0
40–∞	0.8 ± 0.3	0.4 ± 0.1	0.0 ± 0.0	0.0 ± 0.0	0.0 ± 0.0	0.0 ± 0.0
Dark phase						
<0.5	35.0 ± 2.6^**###**^	31.2 ± 2.4^**####**^	6.4 ± 0.8	7.1 ± 0.9	5.8 ± 1.4^**#**^	5.2 ± 1.0^**##**^
0.5–1	9.7 ± 1.2	9.0 ± 1.0	7.8 ± 1.2	7.8 ± 0.8	7.1 ± 0.8	5.6 ± 0.9
1–2.5	5.9 ± 0.9	6.7 ± 0.9	16.7 ± 1.9	17.6 ± 2.0	9.9 ± 1.2^**#**^	10.4 ± 1.6
2.5–5	5.1 ± 1.0	9.2 ± 1.6	21.2 ± 1.5	25.7 ± 2.2	1.1 ± 0.5	1.8 ± 0.6
5–10	6.8 ± 0.9	8.7 ± 1.2	18.1 ± 1.3^**##**^	15.7 ± 1.3^**#**^	0.0 ± 0.0	0.0 ± 0.0
10–20	5.2 ± 0.6	6.1 ± 0.8	3.2 ± 0.8	1.8 ± 0.4^**#**^	0.0 ± 0.0	0.0 ± 0.0
20–40	4.3 ± 0.4^**###**^	3.5 ± 0.5	0.1 ± 0.1	0.0 ± 0.0	0.0 ± 0.0	0.0 ± 0.0
40–∞	1.4 ± 0.2	1.3 ± 0.4	0.0 ± 0.0	0.0 ± 0.0	0.0 ± 0.0	0.0 ± 0.0
Vehicle injection						
<0.5	7.3 ± 2.0	6.5 ± 1.3	2.3 ± 0.6	2.5 ± 0.5	0.7 ± 0.2	1.2 ± 0.3
0.5–1	2.2 ± 0.4	1.4 ± 0.3	2.6 ± 0.9	1.8 ± 0.4	0.7 ± 0.2	1.6 ± 0.4
1–2.5	1.7 ± 0.5	1.0 ± 0.3	4.9 ± 0.7	3.7 ± 0.6	2.3 ± 0.7	2.5 ± 0.4
2.5–5	1.3 ± 0.2	2.4 ± 0.7	4.0 ± 0.8	6.3 ± 0.6	0.0 ± 0.0	0.4 ± 0.3
5–10	2.1 ± 0.6	3.1 ± 0.7	4.5 ± 0.6	3.5 ± 0.6	0.0 ± 0.0	0.0 ± 0.0
10–20	2.3 ± 0.3	2.8 ± 0.5	0.3 ± 0.1	0.3 ± 0.2	0.0 ± 0.0	0.0 ± 0.0
20–40	1.7 ± 0.2	0.9 ± 0.2	0.0 ± 0.0	0.0 ± 0.0	0.0 ± 0.0	0.0 ± 0.0
40–∞	0.4 ± 0.2	0.3 ± 0.2	0.0 ± 0.0	0.0 ± 0.0	0.0 ± 0.0	0.0 ± 0.0
CNO 0.3 mg/kg						
<0.5	6.8 ± 1.1	4.9 ± 1.0	1.6 ± 0.6	1.7 ± 0.6	0.1 ± 0.1	0.4 ± 0.3
0.5–1	3.0 ± 0.4	2.9 ± 0.8	2.6 ± 0.9	2.3 ± 0.5	0.0 ± 0.0	0.5 ± 0.3
1–2.5	2.7 ± 0.7	2.5 ± 0.5	3.8 ± 1.0	3.2 ± 0.9	0.6 ± 0.3	0.2 ± 0.2**^^**
2.5–5	1.6 ± 0.5	1.2 ± 0.5	2.6 ± 0.6	2.2 ± 0.6**^^^**	0.0 ± 0.0	0.0 ± 0.0
5–10	0.9 ± 0.3	2.3 ± 0.4	2.9 ± 0.5	2.4 ± 0.6	0.0 ± 0.0	0.0 ± 0.0
10–20	1.4 ± 0.4	0.6 ± 0.2**^**	1.2 ± 0.6	1.2 ± 0.4	0.0 ± 0.0	0.0 ± 0.0
20–40	0.2 ± 0.1**^^^^**	0.4 ± 0.2	1.0 ± 0.3	0.8 ± 0.3	0.0 ± 0.0	0.0 ± 0.0

**Table 4:** Percentage (± SEM) of wakefulness, slow-wave-sleep, and REM sleep amounts in each bout length to the total amount of wakefulness, slow-wave-sleep, and REM sleep during the light and dark periods: AP+ (*n* = 17) and AP− (*n* = 16) mice; and during the 3-hr following vehicle or CNO injection AP+ (*n* = 10) and AP− (*n* = 11) mice. *p < .05, ***p < .001, ****p < .0001, AP+ vs. AP− mice; ^**#**^*p* < .05, ^**##**^*p* < .01, ^**###**^*p* < .001, ^**####**^*p* < .0001, dark vs. light period; and **^***p* < .05, **^^***p* < .01, **^^^^***p* < .0001 CNO vs. vehicle injection, two-way ANOVA followed by a post hoc Bonferroni test.

Bout length (min)	Wakefulness		Slow-Wave-Sleep		REM Sleep	
Light Phase	AP-	AP+	AP-	AP+	AP-	AP+
<0.5	7.3 ± 0.5	6.5 ± 0.5	0.5 ± 0.1	0.8 ± 0.1	9.8 ± 1.0	8.5 ± 0.7
0.5–1	4.5 ± 0.5	3.7 ± 0.3	1.7 ± 0.2	2.1 ± 0.2	20.0 ± 1.7	14.8 ± 1.2
1–2.5	4.5 ± 0.7	3.8 ± 0.5	9.8 ± 1.2	10.1 ± 1.0	60.2 ± 2.7	55.6 ± 2.4
2.5–5	4.3 ± 0.8	7.1 ± 1.4	25.4 ± 2.3	27.3 ± 1.7	10.1 ± 2.5	21.1 ± 2.8
5–10	12.8 ± 2.1	17.2 ± 2.1	43.5 ± 2.0	43.0 ± 2.2	0.0 ± 0.0	0.0 ± 0.0
10–20	24.2 ± 3.3	30.5 ± 2.7	18.6 ± 2.7	16.4 ± 2.9	0.0 ± 0.0	0.0 ± 0.0
20–40	28.1 ± 3.9	24.1 ± 3.5	0.7 ± 0.5	0.3 ± 0.3	0.0 ± 0.0	0.0 ± 0.0
40–∞	14.4 ± 4.4	7.2 ± 2.6	0.0 ± 0.0	0.0 ± 0.0	0.0 ± 0.0	0.0 ± 0.0
Dark phase						
<0.5	2.9 ± 0.2^**####**^	2.3 ± 0.2^**####**^	0.9 ± 0.1^**#**^	1.1 ± 0.1	7.6 ± 1.4	5.6 ± 0.8
0.5–1	2.0 ± 0.2^**##**^	1.9 ± 0.2^**###**^	2.4 ± 0.4	2.7 ± ± 0.3	22.8 ± 3.1	16.0 ± 2.1
1–2.5	2.6 ± 0.4	2.9 ± 0.5	11.0 ± 1.5	12.2 ± 1.1	59.5 ± 4.4	57.5 ± 3.7
2.5–5	5.1 ± 1.0	8.9 ± 1.6	28.3 ± 2.4	35.7 ± 2.1	10.1 ± 3.9	21.0 ± 5.0
5–10	13.8 ± 1.8	16.2 ± 2.3	44.2 ± 3.1	39.5 ± 2.2	0.0 ± 0.0	0.0 ± 0.0
10–20	19.5 ± 2.3	21.8 ± 3.0	12.9 ± 3.0	8.8 ± 2.3	0.0 ± 0.0	0.0 ± 0.0
20–40	31.6 ± 2.5	24.4 ± 3.8	0.3 ± 0.3	0.0 ± 0.0	0.0 ± 0.0	0.0 ± 0.0
40–∞	22.4 ± 3.2	21.6 ± 6.6	0.0 ± 0.0	0.0 ± 0.0	0.0 ± 0.0	0.0 ± 0.0
Vehicle injection						
<0.5	2.1 ± 0.6	1.9 ± 0.5	1.3 ± 0.3	1.8 ± 0.3	17.5 ± 9.0	16.2 ± 9.5
0.5–1	1.4 ± 0.3	1.0 ± 0.2	3.6 ± 1.1	2.7 ± 0.6	15.9 ± 6.2	18.2 ± 4.5
1–2.5	2.4 ± 0.6	1.4 ± 0.4	16.5 ± 2.6	13.1 ± 2.7	66.6 ± 10.4	56.0 ± 9.2
2.5–5	4.3 ± 0.9	8.0 ± 2.2	24.7 ± 4.3	41.3 ± 3.8***	0.0 ± 0.0	9.6 ± 7.0
5–10	13.5 ± 4.3	22.2 ± 6.2	49.3 ± 5.6	36.6 ± 4.7*	0.0 ± 0.0	0.0 ± 0.0
10–20	27.1 ± 4.3	34.2 ± 5.9	4.5 ± 2.5	4.5 ± 3.2	0.0 ± 0.0	0.0 ± 0.0
20–40	36.6 ± 5.4	19.9 ± 3.7	0.0 ± 0.0	0.0 ± 0.0	0.0 ± 0.0	0.0 ± 0.0
40–∞	12.7 ± 5.4	11.4 ± 8.4	0.0 ± 0.0	0.0 ± 0.0	0.0 ± 0.0	0.0 ± 0.0
CNO 0.3 mg/kg						
<0.5	6.4 ± 1.8	4.5 ± 1.0	0.5 ± 0.2	0.6 ± 0.2**^**	0.6 ± 0.6	15.0 ± 10.7
0.5–1	7.1 ± 1.4**^**	7.0 ± 2.3	2.0 ± 0.6	1.4 ± 0.3	0.0 ± 0.0	19.3 ± 10.9
1–2.5	10.0 ± 2.9	10.0 ± 2.6	6.0 ± 2.0**^**	4.6 ± 1.4	26.7 ± 13.8	5.8 ± 5.8**^^**
2.5–5	13.2 ± 5.3	6.5 ± 2.6	9.5 ± 2.4	6.5 ± 1.8**^^^^**	0.0 ± 0.0	0.0 ± 0.0
5–10	20.0 ± 7.0	43.2 ± 7.1	18.9 ± 3.6**^^**	14.7 ± 3.7**^**	0.0 ± 0.0	0.0 ± 0.0
10–20	31.3 ± 9.2	12.5 ± 3.9	11.8 ± 3.4	11.6 ± 3.3	0.0 ± 0.0	0.0 ± 0.0
20–40	4.1 ± 2.8**^^^^**	12.2 ± 6.8	20.2 ± 5.6**^**	17.7 ± 6.5	0.0 ± 0.0	0.0 ± 0.0
40–∞	8.0 ± 8.0	4.3 ± 4.3	31.1 ± 9.0**^**	43.0 ± 7.8**^^**	0.0 ± 0.0	0.0 ± 0.0

### Statistical analysis

Statistical analysis was performed using Prism v8 (GraphPad Software, San Diego, CA, United States). Following confirmation that the data met the assumptions of the ANOVA model, two-way ANOVA followed by a post hoc Bonferroni test were used to compare the effect of the age, genotype, drug injection or time period on sleep–wake parameters. Paired Student’s *t*-tests were used to compare the effects of the age, genotype or drug injection on the amount of SWS and delta power during the 3-hr period following injection, as well as the SWS and REM sleep latencies following injection.

### Immunolabeling

**Figure 1.**
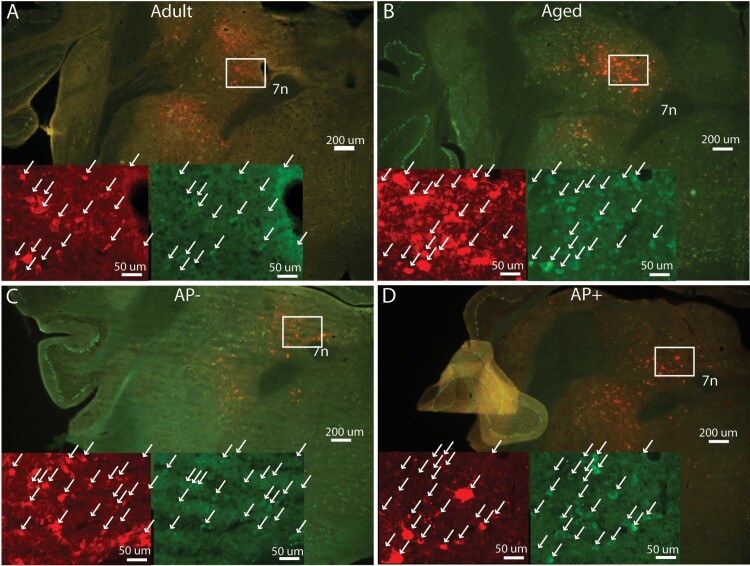
**AAV-hM3Dq-mCherry injection site in PZ.** Coronal sections at the level of PZ showing native green fluorescence of GFP expressing Vgat::GFP neurons and native red fluorescence of hM3Dq-mCherry expressing Vgat::GFP neurons from an adult (A), aged (B), AP- (C) and AP+ (D) mouse. Scale bar = 200 μm. Inserts show high magnification images (scale bar = 50 µm) of the white boxes using native mCherry (red) fluorescence or native green (GFP) fluorescence. White arrows show double labeled neurons. 7n, seventh cranial nerve.

At the end of the experiments, animals were deeply anesthetized with ketamine/xylazine (200 and 20 mg/kg, respectively) and perfused transcardially with 20 mL of saline, followed by 100 mL of neutral phosphate-buffered formalin (4%; Thermo Fisher Scientific). Brains were removed from the skull and incubated in neutral phosphate-buffered formalin for 2-hr, followed by 20% sucrose until they sank, and were subsequently sectioned at 40 μm on a freezing microtome into 3 series. For native fluorescence detection, brain sections from 1 of each series were mounted, coverslipped using ProLong Glass Antifade Mountant (Invitrogen, Cat. # P36984) and visualized with a fluorescence microscope (Keyence BZ-X710, Japan; Figure 1). For amyloid plaque detection, one of each series from AP+ and AP− mice was used. For optimal immunodetection, and to eliminate background staining, we used the Mouse on Mouse (M.O.M.) kit (Catalog# BMK-2202, Vector Laboratories). Floating brain sections were incubated for two nights with primary β-Amyloid antiserum (1:10 000; mouse, catalog # 803014,803017, BioLegend). Afterwards, sections were incubated in M.O.M. antimouse biotinylated secondary antiserum from M.O.M. kit followed by incubation in ABC reagents (1:1000; Vector Laboratories) for 90 min. Visualization of the reaction was in a 0.06% solution of 3,3-diaminobenzidine tetrahydrochloride (Sigma–Aldrich) in PBS plus 0.02% H_2_O_2_ for 15 s to 2 min. Finally, the sections were mounted on slides, counterstained with thionin solution (0.125%), dehydrated, cleared, and coverslipped, and visualized with a brightfield microscope (Keyence BZ-X710, Japan).

## Results

### Altered baseline REM sleep and cortical EEG power distribution in aged mice

Previous studies have shown changes in sleep architecture associated with aging. Age-related changes include a reduction in sleep quality, sleep fragmentation, and circadian disturbances in humans [30]. Similar phenotypes have been described in aged mice [31–34]. To confirm these phenotypes in our experimental conditions, we characterized baseline sleep–wake cycles in aged mice (18–24 months old) as compared with adult mice (3–6 months old).

### Sleep–wake amounts

**Figure 2.**
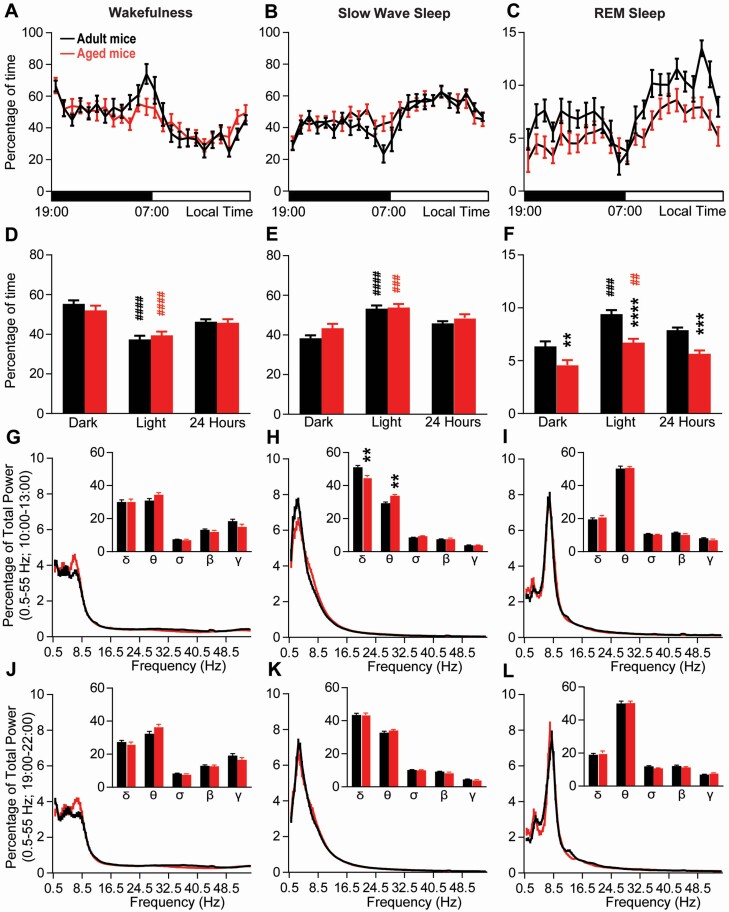
**Baseline sleep-wake cycle in aged vs. adult Vgat::GFP mice.** (**A-C**) Hourly percentage (± S.E.M.) of wakefulness (**A**), slow-wave-sleep (**B**) and REM sleep (**C**). (**D-F**) Percentage (± S.E.M.) of wakefulness (**D**), slow-wave-sleep (**E**) and REM sleep (**F**) during the dark period (19:00–07:00), the light period (07:00–19:00) and 24-hr period. (**G-L**) Cortical EEG power (± S.E.M.) distribution during a time of high sleep drive (10:00–13:00, **G-I**) and a time of low sleep drive (19:00–22:00, **J-L**). Inserts in **G-L**: quantitative changes (± S.E.M.) in power bands: delta (δ, 0.5–4.5 Hz), theta (θ, 4.5–10 Hz), sigma (α, 10–15 Hz), beta (β, 15–30 Hz), gamma (γ, 30–55 Hz). (**A-F**) Adult mice *N* = 17 and aged mice *N* = 17; (G-L) Adult mice *N* = 14 and aged mice N = 12. *p < .05, **p < .01, ***p < .001, ****p < .0001, aged vs. adult mice, two-way ANOVA followed by a post hoc Bonferroni test; ^##^*p* < .01, ^###^*p* < .001, ^####^*p* < .0001 dark vs. light paired Student’s *t*-test.

The age of the mice did not significantly affect the hourly distribution of wakefulness (two-way ANOVA, *F*(1,32) = 0.08, *p* = .78; Figure 2A) or SWS (two-way ANOVA, *F*(1,32) = 1.71, *p* = .20; Figure 2B). Both adult mice and aged mice showed similar daily distributions of wakefulness and SWS with a significantly higher amount of wakefulness during the dark period as compared with the light period (adult mice, *p* < .0001; aged mice, *p* <.0001; Figure 2D). SWS showed the opposite pattern, with higher levels occurring during the light period (adult mice, *p* < .0001; aged mice, *p* < .0001; Figure 2E). Furthermore, the percentage of wakefulness (Figure 2D) and SWS (Figure 2E) were similar between aged mice and adult mice during the light period (07:00–19:00), dark period (19:00–07:00), and 24-hour period. However, REM sleep hourly distribution was significantly affected by age (two-way ANOVA, *F*(1,32) = 30.54, *p* < .0001; Figure 2C). The amount of REM sleep was significantly higher in both adult mice (*p* = .0007) and aged mice (*p* = .001) during the light period as compared with dark period. Also consistent with previous reports, the amount of REM sleep was significantly decreased in aged mice as compared with adult mice during both the light (*p* < .0001) and dark (*p* = .005) periods, resulting in a significant decrease in the daily amount of REM sleep (*p* = .0003; Figure 2F). Therefore, aged mice show a normal amount and distribution of wakefulness and SWS but a decrease in hourly amount of REM sleep.

### Sleep fragmentation

The decrease in REM sleep amount in aged mice was associated with a significant decrease in the number of long duration REM sleep episodes during the light period (1.65 ± 0.34 vs. 6.71 ± 0.53 bouts lasting 2.5–5 min in adult mice, *p* < .0001) and during the dark period (1.35 ± 0.31 vs. 3.88 ± 0.58 bouts lasting 2.5–5 min in adult mice, *p* < .0001; Table 1). In addition, aged mice had a significant decrease in the percentage of REM sleep from long duration bout lengths (9.58 ± 2.02 vs. 34.22 ± 3.26 % of total REM sleep in 2.5–5 min long bout lengths in adult mice; *p* < .0001; Table 2) associated with a significant increase in the percentage of REM sleep from shorter bout durations. The number of wakefulness and SWS episodes, from each bout length, remained unchanged between adult mice and aged mice (Table 1). During the dark period, aged mice exhibited a significant decrease in the percentage of SWS from medium bout lengths (6.64 ± 2.48 vs. 8.46 ± 2.43% of SWS in 10–20 min long bout, in adult mice; *p* = .04; Table 2) which was associated with a trend to increased percentage of SWS from shorter SWS bout lengths in aged mice. Wakefulness bout length distribution remained unchanged, between age groups, in the dark and light period (Table 2). Therefore, aged mice show impairment in the initiation and maintenance of REM sleep. They also show mild SWS fragmentation.

### Cortical EEG power distribution

To further characterize sleep–wake phenotypes in aged mice, we analyzed the cortical EEG power distribution during two time periods, when the mice are mostly asleep (10:00–13:00, ZT3-6) and when the mice are highly awake (19:00–22:00, ZT12-15). During the sleepy period (10:00–13:00), mouse age did not significantly affect the cortical EEG power distribution during wakefulness (two-way ANOVA *F*(1,24) = 0.16, *p* = .69; Figure 2G), SWS (two-way ANOVA *F*(1,24) = 0.43, *p* = .52; Figure 2H), or REM sleep (two-way ANOVA *F*(1,24) = 0.31, *p* = .58; Figure 2I). In wakefulness, individual power bands were similar between aged mice and adult mice (two-way ANOVA *F*(1,24) = 0.26, *p* = .61; Figure 2G, insert). However, SWS delta power was significantly decreased in aged mice compared to adult mice (44.99 ± 1.03 vs. 51.02 ± 1.16% of total power, respectively, *p* = .003) and theta power was significantly increased in aged mice compared to adults (33.86 ± 0.77 vs 29.21 ± 0.87% of total power, respectively, *p* = .003; Figure 2H, insert). These results indicate impaired SWS quality in aged mice during the light period, relative to adult mice. REM sleep individual power bands do not differ between aged and adult mice (two-way ANOVA *F*(1,24) = 0.09, *p* = .77; Figure 2I, insert).

During the active period (19:00–22:00), the cortical EEG power distribution was not significantly affected by mouse age in wakefulness (two-way ANOVA *F*(1,24) = 0.10, *p* = .92; Figure 2J), SWS (two-way ANOVA *F*(1,24) = 0.16, *p* = .69; Figure 2K), or REM sleep (two-way ANOVA *F*(1,24) = 3.15, *p* = .09; Figure 2L). Individual power bands are similar between aged mice and adult mice during wakefulness (two-way ANOVA *F*(1,24) = 3.26, *p* = .08; Figure 2J, insert), SWS (two-way ANOVA *F*(1,24) = 0.02, *p* = .90; Figure 2K, insert), and REM sleep (two-way ANOVA *F*(1,24) = 0.76, *p* = .39; Figure 2L, insert).

Collectively, these data indicate that aged mice show decreased SWS quality (reduced delta power) at the time of the day that SWS amount is the highest (10:00–13:00, ZT3-6; Figure 2H), but not when SWS amount is lower (19:00–22:00, ZT12-15; Figure 2K).

### Chemogenetic activation of PZ^GABA^ powerfully enhances SWS in aged mice to the same extent as adult mice

It has been suggested that decreased SWS quality in aging could be due to the loss of SWS-promoting neurons [35, 36]. Therefore, in this study, we tested whether chemogenetic activation of PZ^GABA^ would be able to elicit enhanced SWS in aged mice, and whether it does so to the same extent as in adult mice. It has previously been shown that chemogenetic activation of PZ^GABA^ results in strong and long-lasting SWS enhancement both when the mice are highly awake (19:00, ZT12) and when they are mostly asleep (10:00, ZT3) [16]. However, the phenotype is more readily apparent when the mice are highly awake. Therefore, in the present study, we injected CNO (or vehicle, in a counterbalanced design) at 19:00.

### Sleep–wake amounts

**Figure 3.**
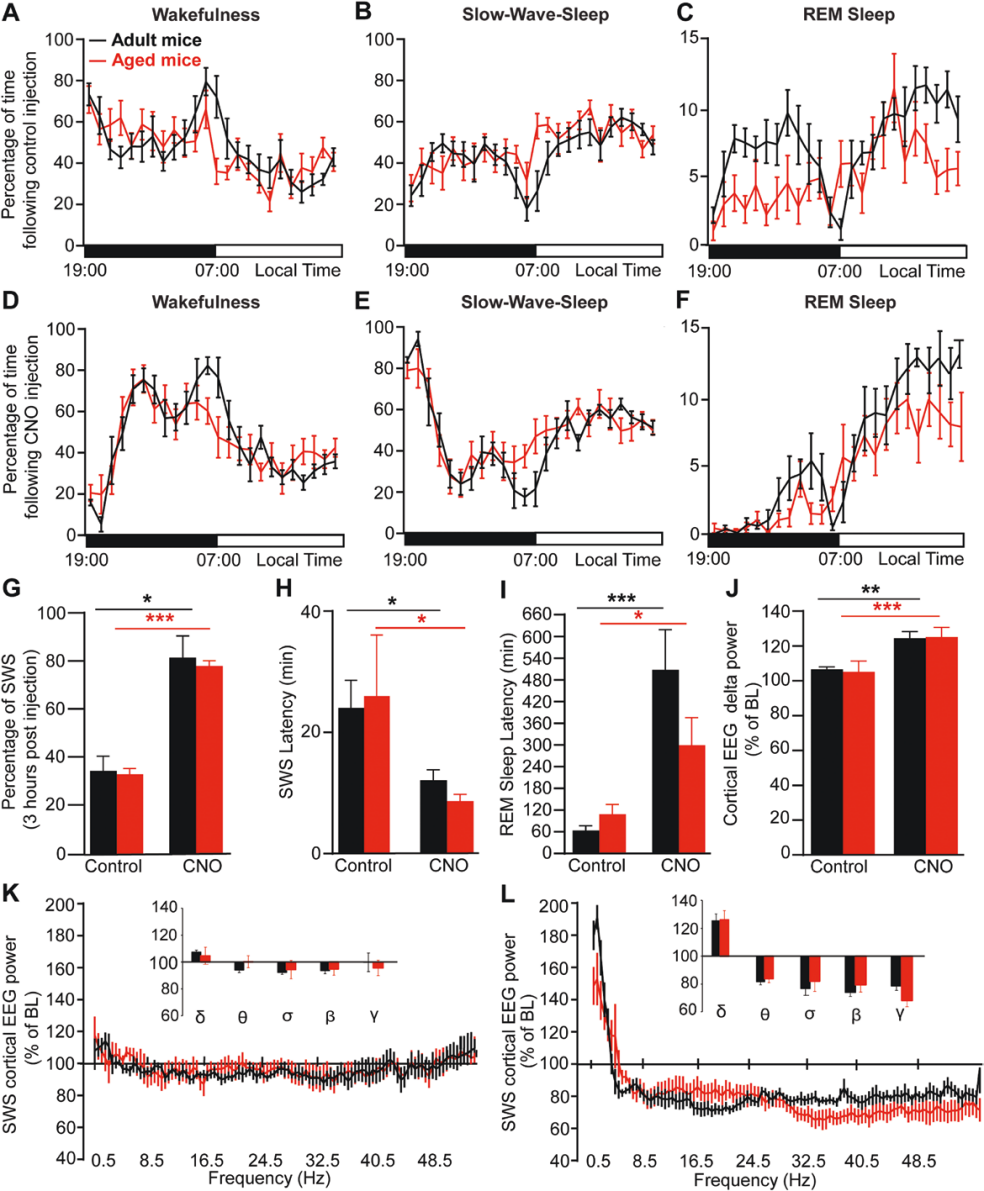
**Chemogenetic activation of PZ GABAergic neurons enhance slow-wave-sleep and slow wave activity in aged mice similar to adult mice.** (**A-F**) Hourly percentage (± S.E.M.) of wakefulness (**A,D**), slow-wave-sleep (SWS; **B,E**) and REM sleep (**C,F**) following control injection (**A-C**) and CNO (0.3 mg/kg) injection (**D-F**). (**G**) Percentage of SWS (± S.E.M.) during the 3-hr post-injection period (ZT12 – 15). (**H**) SWS latency (±S.E.M.). (**I**) REM sleep latency (± S.E.M). (**J**) Cortical EEG delta power (± S.E.M.) during SWS expressed in percentage of baseline (BL) SWS power. (**K-L**) SWS power changes over BL following control injection (**K**) and CNO (0.3 mg/kg) injection (**L**). Inserts in **K-L**: quantitative changes (± S.E.M.) in power bands: delta (δ, 0.5–4.5 Hz), theta (θ, 4.5–10 Hz), sigma (α, 10–15 Hz), beta (β, 15–30 Hz), and gamma (γ, 30–55 Hz). (**A-I**) Adult mice *N* = 8 and aged mice N = 8; (J-L) Adult mice *N* = 7 and aged mice *N* = 7. (**A-F, K-L**) No significant difference between age groups, two-way ANOVA followed by a post hoc Bonferroni test. **(H-J**) *p < .05, **p < .01, ***p < .001, paired Student’s *t*-test.

Following vehicle injection at 19:00, mouse age did not affect the hourly distribution of wakefulness (two-way ANOVA, *F*(1,14) = 0.05, *p* = .83; Figure 3A) or SWS (two-way ANOVA, *F*(1,14) = 0.63, *p* = .44; Figure 3B). However, in accordance with the phenotypes observed in baseline recordings above, REM sleep hourly distribution was significantly affected by the age (two-way ANOVA, *F*(1,14) = 6.81, *p* = .02, Figure 3C). These results indicate that the age does not affect the response to the vehicle injection.

CNO injection (0.3 mg/kg; 19:00, ZT12) was administered to adult mice and aged mice to activate PZ^GABA^. Mouse age did not affect the hourly distribution of wakefulness (two-way ANOVA, *F*(1,14) = 0.05, *p* = .82; Figure 3D), SWS (two-way ANOVA, *F*(1,14) = 0.42, *p* = .53; Figure 3E), or REM sleep (two-way ANOVA, *F*(1,14) = 2.84, *p* = .11; Figure 3F) following CNO, indicating that age did not affect the long-lasting response to chemogenetic activation of PZ^GABA^.

As previously shown [16], in adult mice, CNO injection significantly increased SWS amount during the 3-hr period after injection, compared with vehicle injection (80.80 ± 9.00 vs. 34.20 ± 6.10% of time, respectively, *p* = .04). A similar phenotype was observed in aged mice (77.40 ± 2.20 vs. 32.90 ± 2.30% of time after vehicle injection, *p* = .005; Figure 3G). Importantly, SWS amounts were not significantly different between aged mice and adult mice in the 3-hr period after injection (32.90 ± 2.30 vs. 34.20 ± 6.10% of time in adult mice, *p* = .86) or CNO injection (77.40 ± 2.20 vs. 80.80 ± 9.00% of time in adult mice, *p* = .74). Thus, age does not affect the quantity of SWS induced by the activation of PZ^GABA^.

### PZ^GABA^ activation and bout lengths

In this study, the phenotype of adult mice receiving CNO injections leading to PZ^GABA^ activation recapitulated previous findings [16]. CNO injection (1) significantly increased the number of SWS episodes within long bout lengths (40–∞ min long bouts, *p* < .00001, Table 1) compared to vehicle injection; (2) significantly decreased the percentage of SWS occurring in short bout lengths (1–2.5 min long bouts, *p* = .03 and 2.5–5 min long bouts, *p* = .002) and in medium bout lengths (5–10 min long bouts, *p* = .01, Table 2); and (3) significantly increased the percentage of SWS in long bout lengths (40–∞ min long bouts; *p* = .001; Table 2). Similarly, in aged mice, when compared to vehicle injection, CNO injection significantly decreased the number of SWS episodes in medium bout lengths (2.5–5 min long bouts; *p* = .001) and significantly increased the number of long bout lengths (20–40 min long bouts, *p* = .01; and 40–∞ min long bouts, *p* = .02; Table 1). Furthermore, in aged mice, after CNO injection, the percentage of SWS in medium bout lengths was decreased (2.5–5 min long bouts, *p* = .0001) and the percentage of SWS in long bout lengths was increased (20–40 min long bouts, *p* = .001 and 40–∞ min long bouts, *p* = .03; Table 2), as compared to vehicle injections. These results show that aged mice will respond to activation of PZ^GABA^ with long bouts of SWS, as seen in adult mice.

### Sleep latency

Chemogenetic activation of PZ^GABA^ is characterized by a short SWS latency and delayed REM sleep latency in adult mice [16], as also seen here (Figure 3H-I). We therefore analyzed these parameters in aged mice after activation of PZ^GABA^. After CNO injection, aged mice displayed a significantly shorter SWS latency compared to vehicle injection (8.60 ± 1.10 vs. 26.00 ± 10.10 min, respectively, *p* = 0.02). Importantly, age did not affect SWS latency following either vehicle injection (26.00 ± 10.10 in aged mice vs. 24.00 ± 4.60 min in adult mice, *p* = .86) or CNO injection (8.60 ± 1.10 in aged mice vs. 12.00 ± 1.70 min in adult mice, *p* = 0.12; Figure 3H). In addition, CNO injection significantly lengthened REM sleep latency in both adult mice (503.30 ± 110.50 vs. 62.30 ± 12.80 min following vehicle injection, *p* = .0008) and aged mice (298.00 ± 75.00 vs. 107.00 ± 27.10 min following vehicle injection, *p* = .03). And there was no significant difference between aged and adult mice following either vehicle injection (107.00 ± 27.10 in aged mice vs. 62.30 ± 12.80 in adult mice, *p* = .16) or following CNO injection (298.00 ± 75.00 in aged mice vs. 503.30 ± 110.50 min in adult mice, *p* = .14; Figure 3I). These results show that age does not affect the latency to sleep following activation of PZ^GABA^.

### Cortical EEG power distribution

In adult mice, another typical phenotype characteristic of chemogenetic activation of PZ^GABA^ is enhanced cortical delta power [16]. Since aged mice displayed reduced SWS delta power as compared with adult mice during the sleepy period (10:00–13:00; Figure 2H), we asked if chemogenetic activation of PZ^GABA^ could enhance delta power in aged mice. In fact, age did not affect the cortical EEG power distribution following either vehicle injection (two-way ANOVA, *F*(1,12) = 0.04, *p* = .85; Figure 3K) or CNO injection (two-way ANOVA, *F*(1,12) = 1.15, *p* = .30; Figure 3L). As previously shown [16], delta power significantly increases in adult mice following CNO injection, relative to vehicle injection (Figure 3J). A similar phenotype was observed in aged mice with a significant increase in delta power following CNO injection as compared with vehicle injection (126.10 ± 6.70 vs. 105.90 ± 6.26% of baseline power following vehicle injection, *p* = .0006, Figure 3J). Importantly, the increase in delta power was similar in aged mice as compared with adult mice (126.10 ± 6.70 vs. 125.40 ± 4.88% of baseline power, respectively, *p* = .95, Figure 3J). This indicates that chemogenetic activation of PZ^GABA^ can enhance the cortical EEG delta band in aged mice to a similar extent as in adult mice.

Together, these results show that SWS can be increased and SWA enhanced in aged mice by chemogenetic activation of PZ^GABA^, and this sleep enhancement is comparable to that seen in adult mice.

### AP+ mice display age-dependent accumulation of amyloid plaques in the cortex and hippocampus

**Figure 4.**
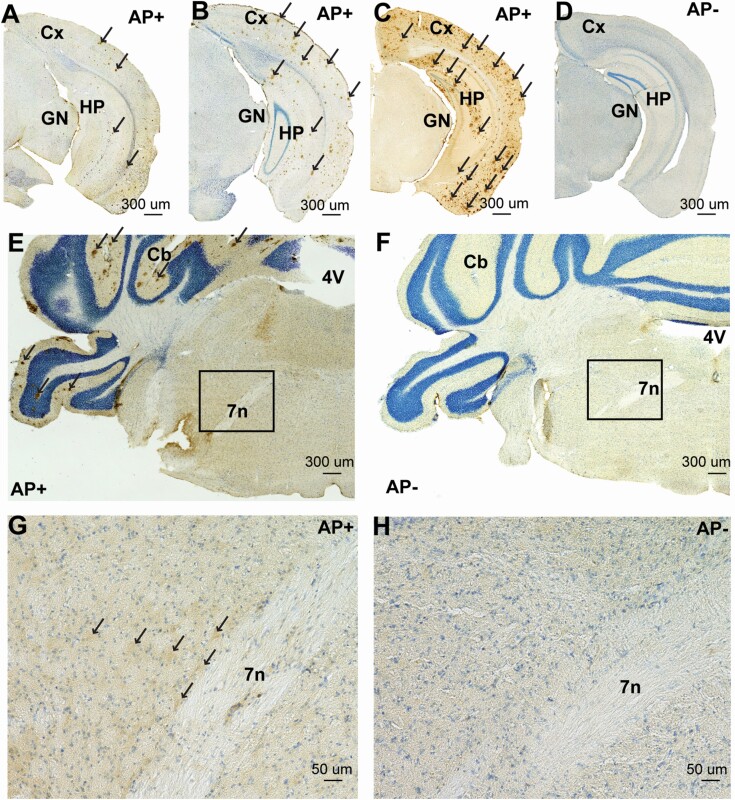
**Amyloid plaques in APP/PS1/Vgat::GFP (AP+) mice and their absence in littermate WT/Vgat::GFP (AP-) mice.** (**A-D**) Amyloid-β labeling (brown) showing amyloid plaques (arrow) in the cortex and hippocampus from 5 month-old (**A**), 10 month-old (**B**) and 14 month-old AP+ mice (**C**). No Amyloid-β labeling is seen in the cortex and hippocampus of a 14 month-old AP- mouse (**D**). (**E-H**) Amyloid-β labeling (brown labeling, arrow) in the parafacial zone from a 14 month-old AP+ mouse (**E,G**) and a 14 month-old AP- mouse (**F,H**). Panels G and H are higher magnification of the box in panels E and F, respectively. Note that amyloid plaques are seen in the cerebellum and higher background labeling is seen in the parafacial zone of the 14 month-old AP+ mouse (**E,G**) as compared with the 14 month-old AP- mouse (**F,H**). 4V, fourth ventricle; 7N, seventh cranial nerve; Cb, cerebellum; Cx, cortex; GN, geniculate nucleus; HP, hippocampus. Scale bar: 300µm (**A-F**), 50µm (**G,H**). Brain slices were counterstained with thionin 0.125% (blue).

To develop a mouse model of SWS/SWA enhancement in AD, we crossed the APP/PS1 mouse line to Vgat::GFP mice, resulting in a quadruple transgenic mouse model (*APP/PS1/*Vgat::GFP; hereafter AP+). We first confirmed that this crossing would not affect the age dependent accumulation and brain distribution of amyloid plaques. AP+ mice display age-dependent accumulation of amyloid plaques in the cortex and hippocampus (Figure 4A–C), with increasing coverage of the cortex and hippocampus with amyloid plaques from 5 to 14 months. As a control, no amyloid immunolabeling was seen in a 14-month-old littermate Vgat::GFP mice lacking APP/PS1 (AP−) mouse (Figure 4D). At the level of the PZ of a 14-month-old AP+ mouse, amyloid deposits are seen in the cerebellum (Figure 4E), yet no amyloid plaques are labeled in other structures, including the PZ. However, in all the inspected PZ, background labeling was always higher in the PZ of AP+ mice (Figure 4G) as compared with the PZ of AP− mice (Figure 4H), suggesting a high level of soluble amyloid-β. These results confirm that the AP+ mouse model retains the main AD phenotype seen in APP/PS1 mice [37]. The presence of amyloid plaques in AP+ mice and their absence in AP− mice was confirmed in all AP mice included in this study, as an additional confirmation of the phenotype.

### Baseline cortical EEG power distribution is affected in AP+ mice

One of the major symptoms of people with AD is impaired sleep–wake behavior, including sleep fragmentation, reduced sleep quality, and circadian disturbances [3]. Similar phenotypes have been described in the APP/PS1 mouse model of AD [38]. Before studying SWS enhancement in these mice, it was important to confirm these phenotypes in our experimental conditions. Hence, baseline sleep–wake cycle was characterized in AP+ mice and compared with littermate control (AP−) mice. Many studies using the APP/PS1 mouse model showed a faster progression of AD phenotypes in female as compared with male mice [39–43]. Moreover, our preliminary analyses indicated that female AP+ mice at the ages of 9–12 months have a similar sleep/wake profile to male AP+ mice aged 12–19 months. The hourly distribution of wakefulness [two-way ANOVA, *F*(1,14) = 0.30, *p* = .59], SWS [two-way ANOVA, *F*(1,14) = 0.20, *p* = .67] and REM sleep [two-way ANOVA, *F*(1,14) = 1.42, *p* = .25] was not significantly different between the two mouse groups. Moreover, no significant changes were seen in sleep-wake fragmentation or cortical EEG power distribution between the two mouse groups. This similarity between older males and younger females confirms previous studies showing an accelerated AD phenotype progression in female than in male APP/PS1 mice at the same age [39–43] and could be explained by the differences in amyloid plaque accumulation in the cortex and hippocampus between sexes [44]. Therefore, this study pooled progression-matched data from nine female (9–12 months, 24–42 g), and seven male (14–17 months, 32.5–48.5 g) APP/PS1/Vgat::GFP (AP+), mice based on the similarity of the phenotypes. The littermate AP− were used as controls (8 females and 7 males).

#### Sleep–wake amounts and sleep fragmentation.

**Figure 5.**
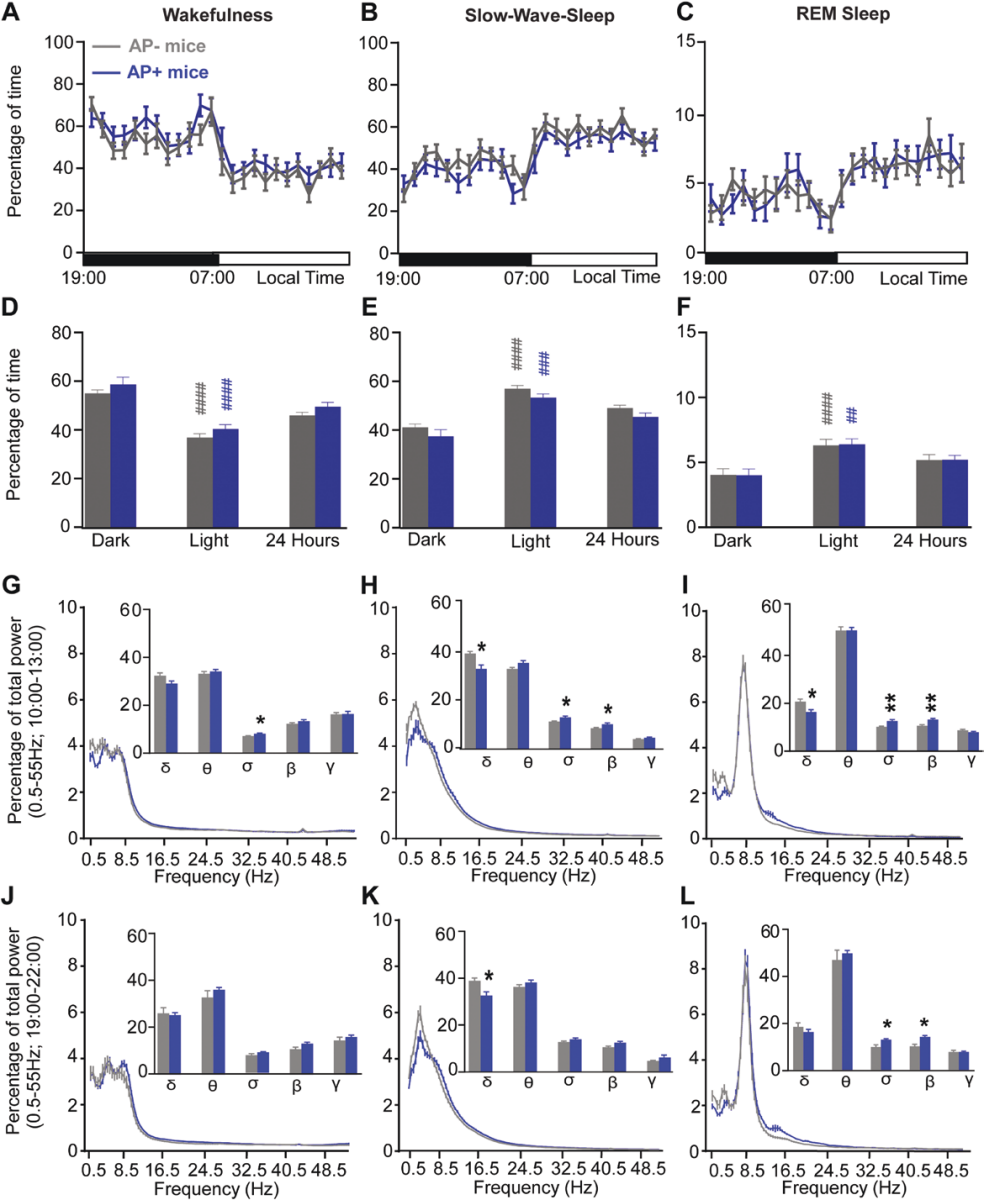
**Baseline sleep–wake cycle in APP/PS1/Vgat::GFP (AP+) mice and littermate wild-type (WT/Vgat::GFP) control (AP-) mice.** Hourly percentage (± S.E.M.) of wakefulness (**A**), slow-wave-sleep (**B**) and REM sleep (**C**). (**D-F**) Percentage (± S.E.M.) of wakefulness (**D**), slow-wave-sleep (**E**) and REM sleep (**F**) during the dark period (19:00–07:00), the light period (07:00–19:00) and 24-hr period. (**G-L**) Cortical EEG power (± S.E.M.) distribution during a time of high sleep drive (10:00–13:00, **G-I**) and a time of low sleep drive (19:00–22:00, **J-L**). Inserts in G-L: quantitative changes (± S.E.M.) in power bands: delta (δ, 0.5–4.5 Hz), theta (θ, 4.5–10 Hz), sigma (α, 10–15 Hz), beta (β, 15–30 Hz), gamma (γ, 30–55 Hz). (**A-F**) AP- mice *N* = 16 and AP+ mice *N* = 15; (G-L) AP- mice *N* = 13 and AP+ mice *N* = 13. *p < .05, **p < .01, AP+ vs. AP- mice, two-way ANOVA followed by a post hoc Bonferroni test; ^##^*p* < .01, ^####^*p* < .0001 dark vs. light period, paired Student’s *t*-test.

AP genotype did not significantly affect the hourly distribution of wakefulness (two-way ANOVA, *F*(1,30) = 2.86, *p* = .10; Figure 5A), SWS (two-way ANOVA, *F*(1,30) = 3.23, *p* = .08; Figure 5B), or REM sleep (two-way ANOVA, *F*(1,30) = 0.002, *p* = .96; Figure 5C). Furthermore, the percentage of wakefulness (Figure 5D), SWS (Figure 5E), or REM sleep (Figure 5F) were similar between AP+ mice and AP− mice during the light period (07:00–19:00), dark period (19:00–07:00), and 24-hour period, indicating no alterations in the daily distribution of sleep–wake amounts. Finally, the number of episodes and percentage of wakefulness, SWS, and REM sleep in various bout lengths were unaffected by the genotype (Tables 3 and 4) during both the light and dark periods. These results indicate that AP+ mice have no deficit in sleep–wake quantity and fragmentation.

#### Cortical EEG power distribution.

Next, we assessed the cortical EEG power distribution of the vigilance stages when the mice are mostly asleep (10:00–13:00) and when the mice are highly awake (19:00–22:00) to investigate putative changes in sleep–wake quality. Importantly, seizures were rarely seen in APP/PS1/Vgat::GFP mice (1 mouse from >50 mice recorded overall in the lab to date), similar to a previous report [29]. Moreover, episodes containing spontaneous epileptiform discharges [29] were excluded from the analysis. Therefore, our power spectral analysis is not affected by adverse events.

During the sleepy period (10:00–13:00), genotype did not significantly affect the cortical EEG power distribution of wakefulness (two-way ANOVA *F*(1,25) = 0.19, *p* = .67; Figure 5G), SWS (two-way ANOVA *F*(1,25) = 1.64, *p* = .21; Figure 5H), and REM sleep (two-way ANOVA *F*(1,25) = 0.009, *p* = .92; Figure 5I). However, in AP+ mice wakefulness was characterized by a significant increase in sigma power (8.08 ± 0.24 vs. 7.08 ± 0.16% of total power in AP− mice, *p* = .01; Figure 5G, insert), as compared with AP− mice. SWS was more affected in AP+ mice compared with AP− mice, with a prominent reduction of delta power (34.34 ± 1.73 vs. 41.05 ± 0.81% of total power, respectively, *p* = .01) associated with a significant increase in both sigma power (13.50 ± 0.57 vs. 11.67 ± 0.27% of total power, respectively, *p* = .05), and beta power (10.56 ± 0.50 vs. 8.82 ± 0.32% of total power, respectively, *p* = .03; Figure 5H, insert), indicating impaired SWS quality in AP+ mice. REM sleep was similarly affected with a significant reduction of delta power (16.29 ± 0.29 vs. 20.57 ± 1.09% of total power in AP− mice, *p* = .03) and a significant increase in both sigma power (12.56 ± 0.56 vs. 10.13 ± 0.30% of total power in AP− mice, *p* = .006) and beta power (13.14 ± 0.46 vs. 10.55 ± 0.41% of total power in AP− mice; *p* = .001; Figure 5I, insert). However, theta power, characteristic of REM sleep in mouse, was not affected (50.21 ± 1.17 vs. 50.10 ± 1.55% of total power in AP+ and AP− mice, respectively, *p* > .1).

During the active period (19:00–22:00), the cortical EEG power distribution was not significantly affected by the genotype during wakefulness (two-way ANOVA *F*(1,25) = 0.93, *p* = .34; Figure 5J), SWS (two-way ANOVA *F*(1,25) = 0.14, *p* = .71; Figure 5K), or REM sleep (two-way ANOVA *F*(1,25) = 0.93, *p* = .34; Figure 5L). No significant changes were observed in individual power bands during wakefulness (Figure 5J, insert). However, during SWS, delta power was significantly decreased in AP+ mice, relative to AP− mice (31.59 ± 1.52 vs. 37.87 ± 1.06% of total power, respectively, *p* = .01; Figure 5K, insert). Compared to AP− mice, REM sleep in AP+ mice was characterized by a significant increase in both sigma power (12.81 ± 0.47 vs. 9.76 ± 0.80% of total power in AP− mice, *p* = .02) and beta power (14.00 ± 0.64 vs. 10.05 ± 0.84% of total power in AP− mice, *p* = .005; Figure 5L, insert). These results show that the quality of the vigilance stages, and specifically of SWS, is affected in AP+ mice during the active period (19:00–22:00) as well as during the sleepy period (10:00–13:00).

### Activation of PZ^GABA^ enhances SWS in AP+ mice to the same extent as littermate AP− mice

It has been suggested that the sleep deficits in AD could result from the loss of sleep-promoting neurons due to the toxicity of amyloid plaque accumulation [2]. Therefore, in this study, we tested whether activation of PZ^GABA^ can enhance SWS in AP+ mice (2 males and 8 females) and compared their response with that of littermate control (AP−) mice (8 males and 3 females).

#### Sleep–wake amounts.

**Figure 6.**
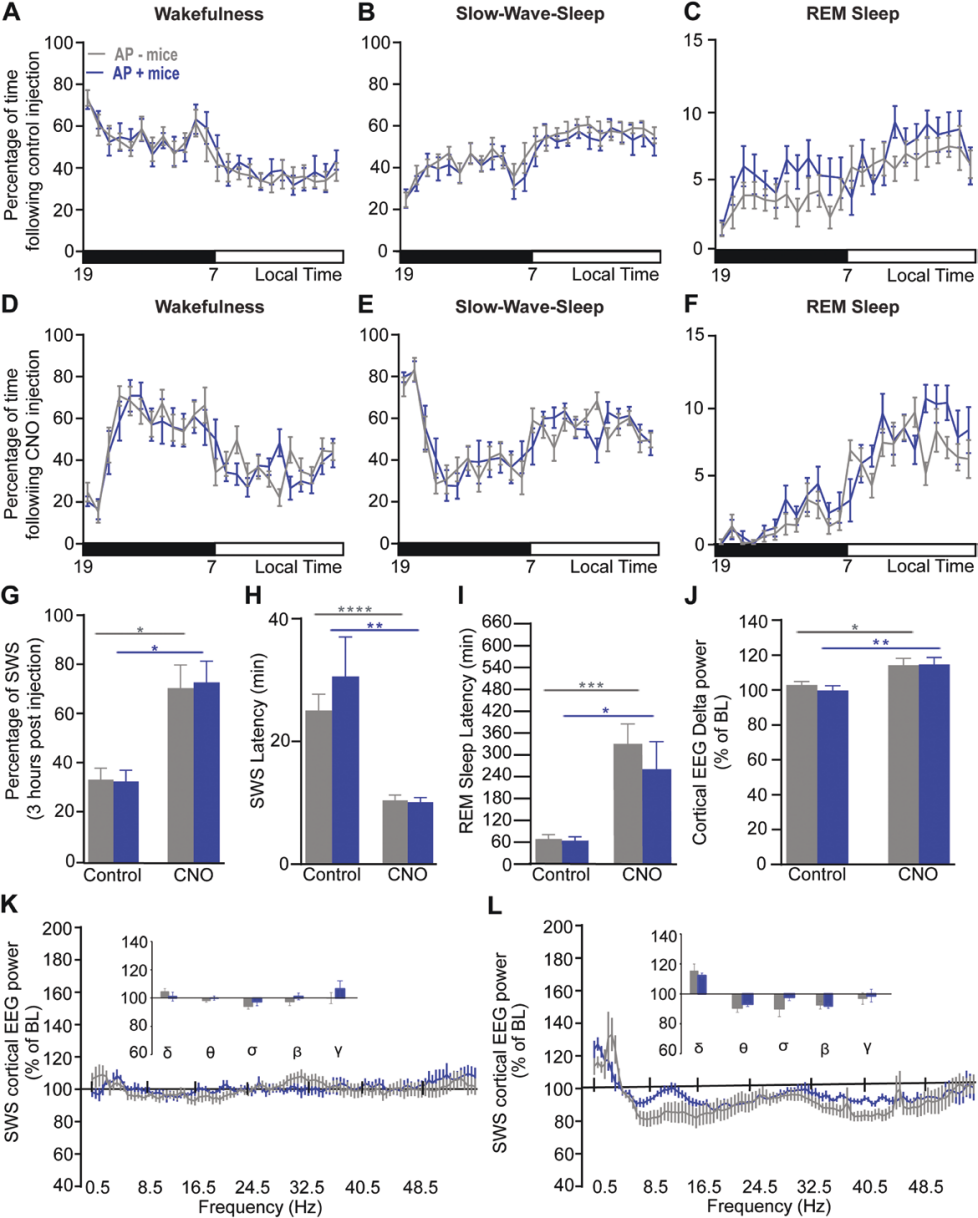
**Chemogenetic activation of PZ GABAergic neurons enhances slow-wave-sleep and slow wave activity in APP/PS1/Vgat::GFP (AP+) mice similar to littermate wild-type (WT/Vgat::GFP) control (AP-) mice. (A-F)** Hourly percentage (± S.E.M.) of wakefulness (**A, D**), slow-wave-sleep (SWS; **B, E**) and REM sleep (**C, F**) following control injection (**A-C**) and CNO (0.3 mg/kg) injection (**D-F**). (**G**) Percentage of SWS (± S.E.M.) during the 3-hr post-injection period (19:00–22:00). **(H)** SWS latency (± S.E.M.). (**I**) REM sleep latency (± S.E.M). (**J**) Cortical EEG delta power (±S.E.M.) during SWS expressed in percentage of SWS baseline (BL) power. (**K-L**) SWS power changes over BL following control injection (**K**) and CNO (0.3 mg/kg) injection (**L**). Inserts in K-L: quantitative changes (± S.E.M.) in power bands: delta (δ, 0.5–4.5 Hz), theta (θ, 4.5–10 Hz), sigma (α, 10–15 Hz), beta (β, 15–30 Hz), gamma (γ, 30–55 Hz). (A-I) AP- mice *N* = 10 and AP+ mice N = 11; (**J-L**) AP- mice *N* = 9 and AP+ mice N = 8. (**A-F, K-L**); No significant changes between AP+ and AP- mice, two-way ANOVA followed by a post hoc Bonferroni test. **(H-J)** *p < .05, **p < .01, ***p < .001, ****p < .0001, paired Student’s *t*-test.

Following vehicle injection at lights-off (19:00), AP genotype did not affect the hourly distribution of wakefulness (two-way ANOVA, *F*(1,19) = 0.28, *p* = .61; Figure 6A) and SWS (two-way ANOVA, *F*(1,19) = 1.07, *p* = .31; Figure 6B). Surprisingly, given that baseline REM sleep daily distribution is not affected by the AP genotype (see previous section), following vehicle injection, REM sleep hourly distribution was affected by the AP genotype (two-way ANOVA, *F*(1,19) = 4.77, *p* = .04; Figure 6C). CNO (0.3 mg/kg; 19:00) was administered to AP+ mice and AP− mice to activate the PZ^GABA^. Following CNO injection, the AP genotype did not affect hourly distribution of wakefulness (two-way ANOVA, *F*(1,19) = 0.20, *p* = .66; Figure 6D), SWS (two-way ANOVA, *F*(1,19) = 0.05, *p* = .83; Figure 6E), or REM sleep (two-way ANOVA, *F*(1,19) = 2.15, *p* = .16; Figure 6F). As previously described in adult mice and aged mice ([16] and above), CNO injection significantly increases SWS amounts during the 3-hr post injection period in AP+ mice as compared with vehicle injection (73.00 ± 8.59 vs. 33.00 ± 4.63% of time after vehicle injection, *p* = .02). A similar increase in SWS amount was observed in AP− mice following CNO injection (70.80 ± 9.32 vs. 33.77 ± 4.65% of time following vehicle injection, *p* = .02; Figure 6G). Importantly, SWS amounts were not significantly different between AP+ mice and AP− mice following either vehicle injection (33.00 ± 4.63 vs. 33.77 ± 4.65% of time in AP− mice, *p* = .91) or CNO injection (73.0 ± 8.59 vs. 70.80 ± 9.32% of time in AP− mice, *p* = .87, Figure 6G). These results indicate that the AP+ genotype and associated central nervous system (CNS) morbidities do not affect the ability of PZ^GABA^ activation to promote SWS.

#### Sleep latency.

Chemogenetic activation of PZ^GABA^ is characterized by a short SWS latency and increased REM sleep latency in adult mice and aged mice ([16] & Figures 3H-I & 16). We therefore analyzed these parameters in AP+ mice and AP− mice. After CNO injection, SWS latency was significantly shorter than after vehicle injection in both AP+ mice (10.02 ± 0.75 vs. 30.43 ± 6.40 min, respectively, *p* = .006) and AP− mice (10.30 ± 0.90 vs. 24.92 ± 2.63 min, respectively, *p* < .0001; Figure 6H). Of note, the AP genotype did not affect SWS latency after vehicle injection (30.43 ± 6.40 vs. 24.92 ± 2.63 min in AP+ and AP− mice, respectively, *p* = .40) or CNO injection (10.02 ± 0.75 vs. 10.30 ± 0.90 min in AP+ and AP− mice, respectively, *p* = .82; Figure 6H). In addition, CNO injection significantly increased REM sleep latency in both AP+ mice (260.40 ± 75.88 vs. 65.06 ± 10.42 min following vehicle injection, *p* = .02, Figure 6I) and AP− mice (330.10 ± 54.85 vs. 69.42 ± 12.21 min following vehicle injection, *p* = .0002, Figure 6I). The AP genotype did not affect REM sleep latency following either vehicle injection (65.06 ± 10.42 vs. 69.42 ± 12.21 in AP− mice, *p* = .79; Figure 6I) or CNO injection (260.40 ± 75.88 vs. 330.10 ± 54.85 min in AP− mice, *p* = .46; Figure 6I). These results show that the AP+ genotype and associated CNS morbidities [37] do not affect the latency to sleep following the activation of PZ^GABA^.

#### Sleep fragmentation.

Following either CNO or vehicle injection, wakefulness, SWS and REM sleep episode numbers did not differ significantly between AP+ mice and AP− mice (Table 3). However, following vehicle injection, as compared to AP− mice, AP+ mice showed a significant increase in the percentage of SWS from medium duration bout lengths (41.30 ± 3.80 vs. 24.70 ± 4.30 at 2.5–5 min long bouts, *p* = .0003), associated with a decrease in the percentage of SWS from slightly longer bout lengths (36.60 ± 4.70 vs. 49.30 ± 5.60 at 5–10 min long bouts, *p* = .01; Table 4). This is consistent with the trend observed in baseline condition, during the dark period. As described above in adult mice and aged mice, in AP- mice, CNO injection: (1) significantly increased the number of SWS episodes in long bout lengths (20–40 min long bouts, *p* = .03; Table 3); (2) significantly decreased the percentage of SWS from short bout lengths (1–2.5 min long bouts, *p* = .003) and medium bout lengths (5–10 min long bouts, *p* = .002, Table 4); and (3) significantly increased the percentage of SWS from long bout lengths (20–40 min long bouts, *p* = .04; 40–∞ min long bouts, *p* = .05; Table 4) as compared to vehicle injection.

Similarly, in AP+ mice, CNO injection significantly decreased the number of SWS episodes from medium bout lengths (2.5–5 min long bouts, *p* = .0008) and increased the number of SWS episodes from long bout lengths (20–40 min long bouts, *p* = .006) compared to vehicle injection (Table 3). CNO injection significantly decreased the percentage of SWS from short bout lengths (0.1–1 min long bouts, *p* = .03) and medium bout lengths (2.5–5 min long bouts, *p* < .0001 and 5–10 min long bouts, *p* = .01). This was associated with a significant increase in the percentage of SWS from long-bout lengths (20–40 min long bout lengths, *p* = .003) as compared to vehicle injection (Table 4). Together, these results suggest that activation of PZ^GABA^ can consolidate SWS similarly between AP+ mice and AP− mice.

#### Cortical EEG power distribution.

We previously showed that chemogenetic activation of PZ^GABA^ increases cortical EEG delta power in both adult mice and aged mice ([16] and Figure 3J). Because in baseline SWS, the cortical EEG delta power is decreased in AP+ mice vs. AP− mice (Figure 5H, K inserts), we tested if activation of PZ^GABA^ can enhance delta power in AP+ mice to the same extent as AP− mice. Our data show that the AP genotype did not affect the cortical EEG power distribution following either vehicle injection (two-way ANOVA, *F*(1,16) = 0.10, *p* = .76) or CNO injection (two-way ANOVA, *F*(1,16) = 1.06, *p* = .32; Figure 6K-L). The cortical EEG delta power was robustly increased after CNO injection as compared with vehicle injection in both AP+ mice (115.90 ± 4.00 vs 100.70 ± 2.810% of baseline power in vehicle condition, *p* = .01) and AP− mice (115.30 ± 4.15 vs 104.10 ± 2.00% of baseline power in vehicle condition, *p* = .04, Figure 6J). Importantly, the AP genotype did not affect the cortical EEG delta power following either vehicle injection (100.70 ± 2.81 vs. 104.10 ± 2.00% of total power in AP− mice, *p* = .37) or CNO injection (115.90 ± 4.00 vs. 115.30 ± 4.15% of total power in AP− mice, *p* = .92; Figure 6J). These results show that the AP+ genotype does not affect SWS delta power following the activation of PZ^GABA^.

Collectively, these results show that AP+ mice respond to chemogenetic activation of PZ^GABA^ in a manner similar to AP− mice. Therefore, SWS can be effectively enhanced in AP+ mice.

### CNO injection does not affect SWS quantity and quality or sleep latencies in Cre− aged and AP+ mice

**Figure 7.**
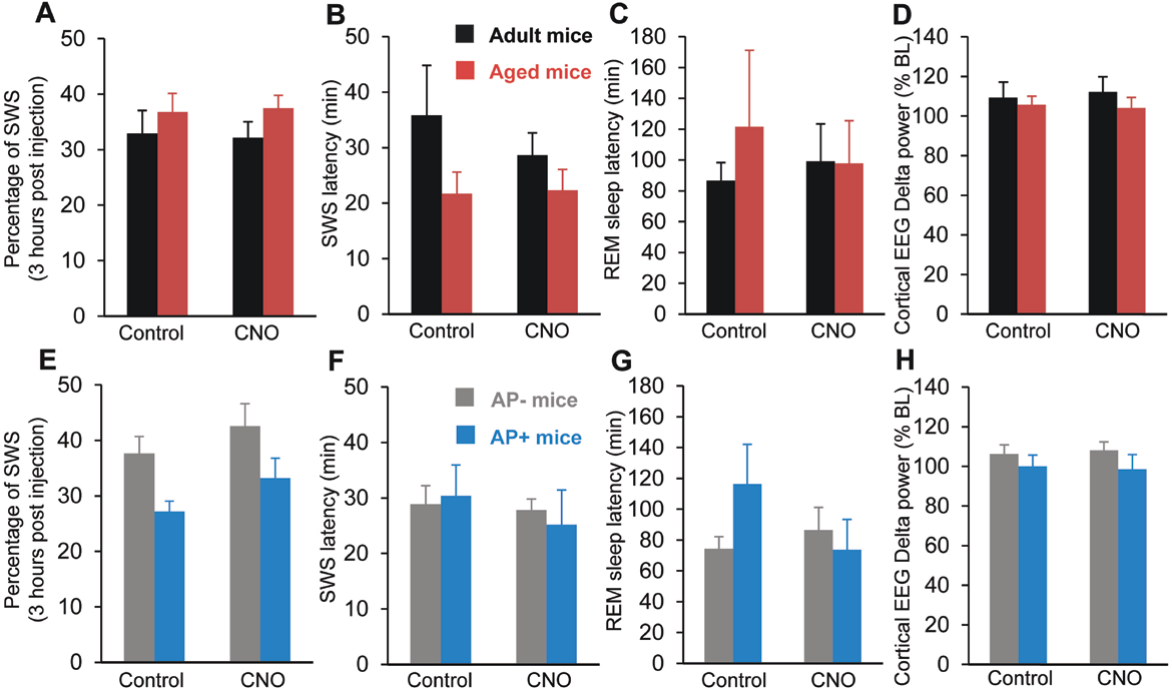
**CNO injection in Cre- littermate control does not affect SWS amount and delta power or sleep latencies.** (**A-D**) Adult and aged Cre- mice. (**E-H**) AP- and AP+ Cre- mice. (**A, E**) Percentage of SWS (± S.E.M.) during the 3-hr post-injection period (19:00–22:00). No significant changes between control and CNO injection, two-way ANOVA followed by a post hoc Bonferroni test. **(B,F)** SWS latency (± S.E.M.). No significant changes between control and CNO injection, paired Student’s t-test. (**C,G**) REM sleep latency (± S.E.M). No significant changes between control and CNO injection, paired Student’s t-test. (**D,H**) Cortical EEG delta power (±S.E.M.) during SWS expressed in percentage of SWS baseline (BL) power. (**A-C**) Adult mice *N* = 8 and aged mice *N* = 9; (**D**) Adult mice *N* = 8 and aged mice *N* = 6. (**E-G**) AP- mice *N* = 9 and AP+ mice *N* = 7; (**H**) AP- mice *N* = 8 and aged mice *N* = 6. No significant changes between control and CNO injection, two-way ANOVA followed by a *post hoc* Bonferroni test.

In a previous study, we showed that, at the dose of 0.3 mg/kg, CNO does not affect sleep–wake quantity or quality in adult mice that do not express the chemogenetic receptor hM3Dq [16]. Here, we confirm the absence of nonspecific effects on sleep of CNO in aged and AP+ mice. Similar to adult mice (*N* = 8), in aged (*N* = 9), AP− (*N* = 5 male and 4 female) and AP+ (*N* = 3 male and 4 female) mice the quantity of SWS during the 3-hr postinjection period is not affected by CNO (0.3 mg/kg) injection, as compared with control injection (Figure 7A,E). Similar results were obtained for SWS and REM sleep latencies (Figure 7B,C, F,G) as well as for the cortical EEG delta power during SWS (Figure 7D,H). These results confirm that in aged, AP− and AP+ mice, CNO (0.3 mg/kg) does not display nonspecific actions on sleep–wake parameters. Therefore, the SWS enhancement phenotype previously described in aged, AP− and AP+ mice expressing hM3Dq receptors in PZ^GABA^ is specific to chemogenetic activation of PZ^GABA^.

## Discussion

Sleep plays a crucial role in homeostasis throughout the body, but particularly in the brain. During human aging as well as in numerous neurological conditions, the quality of deep sleep (SWS) is markedly reduced [45]. Therefore, characterizing the physiological role of SWS is critical in developing strategies designed to reduce the burden of aging and prevent diseases [10, 46]. A previous study has established a mouse model of SWS/SWA enhancement in adult mice which provides a pivotal tool to trigger SWS with high SWA using chemogenetic activation of the PZ [16]. This model is uniquely suited to characterize the role of SWS/SWA in physiology and diseases. In this present study, we show that chemogenetic activation of PZ^GABA^ enhances SWS in aged and AP+ mice to the same extent as in adult and AP− mice, respectively. These results provide new mouse models in which SWS can be induced and enhanced on demand, and this in turn now opens the door to specifically study the role of SWS in physiology and disease, using, for the first time, gain of SWS experiments.

### Sleep–wake phenotypes associated with aging

In the present study, we show that, relative to adult mice, aged mice display reduced 24-hr REM sleep amounts, a trend toward SWS fragmentation and reduced SWA during SWS. These results are in accordance with multiple studies [32, 34, 47, 48] describing similar sleep–wake impairments in aged mice. Some studies have shown an increase in the daily amount of SWS associated with a decrease in the daily amount of wakefulness in aged mice, as compared with adult mice [32, 47, 48]. In this study, no significant changes are seen in the daily amount of both wakefulness and SWS. Our results replicate the finding from Hasan et al. [34] showing no change in daily SWS amounts between 3-month old and 2-year old C57BL/6 mice. Aged mice also display a trend toward SWS fragmentation compared with adult mice with a significant decrease in the percentage of SWS occurring in long bout lengths during the dark period, a phenotype that has been previously reported [48]. Though SWS power density was scored in 4 s epochs, it should be noted that sleep–wake quantity was scored in 10 s epochs and therefore this analysis leaves out short arousal lasting less than 10 s. Scoring sleep-wake quantity in 4 s epochs would have increased total sleep fragmentation in both adult and aged mice. We have been using 10 s epochs for sleep–wake studies to mirror sleep fragmentation studies in humans. Indeed, human sleep-wake scoring is performed in 30 s epochs and the assigned epoch stage is the stage comprising the longest portion of the epoch when two or more stages coexist in a single epoch [26]. Therefore, in humans, sleep fragmentation studies do not include short arousals. Some studies have specifically studied micro-arousals [49, 50] but as far as we are aware, no such studies have been conducted in aging and AD sleep.

SWS quality is most consistently shown to be impaired with aging. Specifically, the percentage of deep NREM sleep is reduced [5]. In mice, it is extremely difficult to distinguish light NREM sleep from deep NREM sleep and typically only one NREM sleep stage, called SWS in our study, is scored. Therefore, measurement of NREM sleep depth in mice is achieved by measuring SWA during SWS. We show that when the mice are sleeping the most, indicating highest sleep pressure (10:00–13:00), the SWS cortical EEG delta (0.5–4.5 Hz) power (also called SWA) is reduced in aged mice compared to adult mice. However, this phenotype is not seen at the beginning of the dark period, when the mice are highly awake and so sleep pressure is at its nadir. These results are in accordance with studies showing reduced daily variations in SWA [34, 48]. Other studies have suggested an increase in SWA with aging in mice. However, the difference in recording and analysis methodologies makes it difficult to compare with our results. For instance, Soltani et al. (2019) shows an increase in SWA obtained from fronto-cerebellar derivations but not from parieto-cerebellar derivations [33]. In this study, the cortical EEG was obtained from fronto-parietal derivations, which could explain the difference in our findings. Panagiotou et al. (2017) filtered and analyzed the slow waves only, and found an increase in absolute SWA with age [32]. In our study, SWA from all SWS bouts was analyzed as a percentage of total power, permitting measurement of overall SWS depth and not sporadic SWA.

REM sleep deficits are also consistently described in aging. Consistent with previous studies [34, 47], we show here that REM sleep amount is affected by age with a reduction of REM sleep amount during the light, dark and 24-hour periods. The decrease in REM sleep is due to a significant decrease in the number of REM sleep episodes and a decrease in the duration of REM sleep episodes. These results indicate a deficit in both initiation and maintenance of REM sleep in aged mice.

### A mouse model of SWS enhancement in aging

We show that chemogenetic activation of PZ^GABA^ powerfully increases SWS amount and enhances SWA, to the same extent as in adult mice. Specifically, SWS amount is increased during the 3-hr period following CNO injection, SWS latency is very short and the cortical EEG delta power is increased, as previously shown in adult mice [16]. Importantly, REM sleep and wake are considerably inhibited, and this allows for the mechanistic study of the effect of only one sleep stage, SWS.

The similar SWS enhancement phenotypes between adult and aged mice is of particular relevance. Our ability to effectively enhance SWS in aged mice by activation of PZ^GABA^ indicates this neuronal population may be a therapeutic target for treating sleep loss in human aging. It has also been suggested that, during human aging, the thinning of cortical gray matter could be responsible for the decreased SWA during SWS [21]. This hypothesis is supported by multiple studies showing a strong relationship between gray matter volume and SWA during development in adolescents [51, 52]. White matter regions and the corpus callosum in particular have also been implicated in SWA intensity and in the propagation of the slow waves between hemispheres in humans [53, 54]. Interestingly, in mice, local cortical neural dynamics and local sleep homeostatic mechanisms seem to be preserved during aging [55]. In the present study, the well-preserved SWS/SWA enhancement in aged mice as compared with adult mice also indicates that the neuronal substrate for SWS and SWA is preserved during aging in mice. Studies in humans showing that SWS/SWA can be enhanced in elderly patients [10] indicate similar aging mechanisms between mouse and human and indicate that mice are good models to study the impact of aging in sleep control.

Multiple studies have shown that SWS/SWA can be enhanced in older adults using physical exercise, transcranial electrical stimulation, transcranial magnetic stimulation and sensory stimulation with positive outputs on cognition [10]. However, studies in humans can only correlate the amount and quality of sleep to physiological outcomes. To study the mechanisms by which SWS/SWA influences physiology, animal models are needed. However, in animal models, and in mice in particular, SWS/SWA enhancement is difficult. Sleep medications that enhance SWS/SWA in humans, such as sodium oxybate, do not restrict the sleep stage to SWS, and they also fail to induce physiological SWS/SWA in rodents [56, 57]. In the present study, we validate the first mouse model of SWS/SWA enhancement in aged mice, thus opening large avenues for investigating the role of SWS/SWA in physiology, in the context of aging.

### Sleep–wake and amyloid phenotypes associated with AD

The goal of this study was to validate a mouse model of SWS/SWA enhancement in AD, necessary for studying the role of SWS/SWA in AD progression and pathology. To study AD mechanistically, numerous animal models are available. In this study, we chose the APP/PS1 mouse model, which has the advantage of recapitulating the sleep deficits observed in humans [38, 58, 59]. More importantly, in this mouse model, the sleep and cognitive deficits appear at mid-age, concomitant to the accumulation of amyloid plaques [60], indicating that the development of the disease, and not over-expression of the transgenes, is responsible for the deficits. To develop a mouse model of SWS/SWA enhancement in AD, we crossed the APP/PS1 mouse line with the Vgat::GFP mouse line. All evidence indicates that this crossing does not affect either the sleep or AD phenotypes described for the original lines: AP+ mice showed age-dependent accumulation of amyloid plaques, the baseline sleep–wake phenotypes are similar to those previously described in the literature in APP/PS1 mice, and PZ^GABA^ seems to not be affected by amyloid burden.

We confirmed the presence of amyloid plaques in the cortex and hippocampus of AP+ mice and their absence in AP− littermate controls. Importantly, as previously shown [37] amyloid plaques were predominantly seen in the cortex, hippocampus and cerebellum. No plaques were seen in other subcortical areas even though the background labeling is consistently higher in AP+ brains as compared with AP− brain, including in the PZ, suggesting higher levels of soluble amyloid-β.

Consistent with findings in APP/PS1 mice [61], the daily distribution of the vigilance stages was unaffected in AP+ compared to AP− mice. No sleep fragmentation was observed. The age-dependent impairment of sleep–wake quantity in APP/PS1 mice is controversial, as some studies show an age dependent increase in wakefulness at the expense of both NREM sleep and REM sleep [58, 62]. Discrepancies in the results might be due to the mouse genetic background and/or the experimental conditions. Importantly, similar to previous reports [38, 61, 63], in our study AP+ mice display a deficit in SWS depth characterized by a decrease in SWA. In accordance with Kent et al. [38], but in contrast with Wang et al. [64], our results do not show changes in theta power across vigilance stages.

### A mouse model of SWS enhancement in AD

Next, we sought to determine whether SWS can be enhanced in AP+ mice to the same extent as AP− mice. Indeed, in AD, it has been suggested that the decrease in SWS quality is due to neurodegeneration in brain regions that control sleep [65]. Accumulation of amyloid plaques in the cortex may also affect cortical synchronization and reduce SWA during SWS [66]. Following chemogenetic activation of PZ^GABA^, AP+ mice show SWS enhancement indistinguishable from AP− mice. Both SWS amount and cortical EEG delta power are enhanced during the 3-hr period following CNO injection, in AP+ mice to the same extent as AP− mice, providing a new mouse model of SWS/SWA enhancement in AD.

A general consensus in the field of Alzheimer’s research posits a critical role of sleep in the progression and in the symptoms of AD. Insufficient sleep could be an easily modifiable and treatable risk factor to reverse symptoms of the disease [11, 12]. Good sleep quality could limit the accumulation of toxic metabolites in the brain. Multiple studies have shown that, during wakefulness, amyloid-β accumulates in the CSF and interstitial fluid, and tau propagation is increased, due to increased neuronal activity [67–69]. During SWS, amyloid-β is cleared by the glymphatic system [62, 70] and tau propagation is suspected to be limited due to decreased neuronal activity. Interestingly, SWA seems the most beneficial component of sleep in AD. Enhancing cortical slow oscillations in humans using transcranial stimulation has been shown to have a positive impact on cognition [71]. Increased amyloid-β accumulation has been specifically correlated with SWA disruption and not with sleep time or efficiency in human [72]. In animals, enhancing cortical SWA continuously for weeks, using optogenetic driving of excitatory cortical neurons reduces the amyloid burden and inhibits other AD markers in APP/PS1 mice [73], whereas chronic sleep deprivation in AD mice accelerates amyloid-β accumulation in the brain and worsens the cognitive deficits [74, 75]. However, all these observations are correlative and the mechanism by which SWS and SWA slow down the progression of AD and reduce the burden of AD remains a mystery. Our mouse model of SWS/SWA enhancement in AD is a new model that will allow to study mechanistically the relationship between sleep and AD.

### Limitations of the models

Our preliminary statistics comparing AP males and AP females indicated that SWS enhancement is not affected by sex. However, in our aging study only male adult and aged mice were used, leaving open the question of an effect of sex in the phenotypes described. Historically, most of the studies on sleep and aging report findings in male mice [32, 55, 76], and when female mice are included in the study, no direct comparison are made between male and female phenotypes [77]. Since the present study examined whether the SWS enhancement model described previously in male mice [16] is functional in aged mice, we chose males for this study. However, given the differences between men and women during aging, including in sleep architecture [78], and the fact that women live longer than men [79], studying the effect of age in female animal models is critical and will be pursued in future studies.

The APP/PS1 mouse model not only has been extensively used to study AD symptoms/mechanisms but also presents some limitations that are important to discuss [80]. The APP/PS1 mouse model, like all mouse models of AD [80], does not completely recapitulate AD pathophysiology. In this case, while AD is characterized by not only accumulation of amyloid plaques in the brain but also of neurofibrillary tangles (NFTs) resulting from aggregates of hyperphosphorylated tau protein [81], the APP/PS1 mouse model lacks these NFTs. On the other hand, the APP/PS1 mouse model does have the advantage of recapitulating most of the deficits associated with AD in humans, such as sleep deficits [38], cognitive impairment [82], and inflammation [83], phenotypes that are also seen when sleep is impaired [84]. In recent years, AD research has increasingly emphasized the relationship between sleep and AD. For instance, a bi-directional relationship between sleep and AD has been suggested, in which lifelong sleep deficits are a risk factor for developing AD, and AD neuropathology in nuclei regulating sleep–wake cycles worsen sleep disruption [2, 3]. Therefore, our mouse model of SWS enhancement will allow us to test whether improving sleep quality is a successful strategy to reduce the burden of AD and will provide important insight into the mechanism by which SWS influences other physiological functions.

In the present study, we show that SWS can be enhanced acutely in aged and AD mouse models. However, symptoms associated with aging and AD develop over decades in humans [85] and months in mice [44]. Therefore, we can speculate whether acute enhancement of SWS can affect phenotypes that developed over time or if chronic SWS enhancement will be necessary. For example, chronic SWS enhancement over months might be necessary to limit the progression of the disease in APP/PS1 mice. Our unpublished preliminary data indicate that chronic daily chemogenetic activation of PZ^GABA^ neurons results in similar SWS enhancement throughout a 6-month period, suggesting that chronic SWS enhancement may be achievable using our mouse model.

In the present study, chemogenetic activation of PZ^GABA^ neurons is achieved using the chemogenetic agonist CNO. However, recent studies have shown that CNO is metabolized into clozapine which is not pharmacologically inert and results in non-specific effects [86]. We have shown in this study and in previous studies [16] that acute administration of CNO at the dose of 0.3 mg/kg does not affect sleep–wake phenotypes in control mice. However, we have not studied other physiological outcomes. Moreover, chronic administration of CNO is likely to result in accumulation of the metabolite clozapine and nonspecific effects. Recent studies have developed alternative agonists, such as compound 21 (C21) [87] and deschloroclozapine (DCZ) [88]. These agonists seem to not be metabolized into pharmacologically active molecules and are a good alternative for chemogenetic studies.

In conclusion, our study shows, for the first time, that SWS can be enhanced in aged and AP+ mice to the same extent as in adult and AP− mice, respectively. These findings provide new and unique models of SWS sleep enhancement in aging and AD. These models will be useful to study the mechanism of SWS/SWA-dependent functions, using gain-of-SWS experiments.
